# RSR-2, the *Caenorhabditis elegans* Ortholog of Human Spliceosomal Component SRm300/SRRM2, Regulates Development by Influencing the Transcriptional Machinery

**DOI:** 10.1371/journal.pgen.1003543

**Published:** 2013-06-06

**Authors:** Laura Fontrodona, Montserrat Porta-de-la-Riva, Tomás Morán, Wei Niu, Mònica Díaz, David Aristizábal-Corrales, Alberto Villanueva, Simó Schwartz, Valerie Reinke, Julián Cerón

**Affiliations:** 1 Cancer and Human Molecular Genetics, Bellvitge Biomedical Research Institute - IDIBELL, L'Hospitalet de Llobregat, Barcelona, Spain; 2 *C. elegans* Core Facility, Bellvitge Biomedical Research Institute - IDIBELL, L'Hospitalet de Llobregat, Barcelona, Spain; 3 Institute of Molecular Biology of Barcelona, IBMB - CSIC, Parc Científic de Barcelona, Barcelona, Spain; 4 Yale University School of Medicine, New Haven, Connecticut, United States of America; 5 Drug Delivery and Targeting, CIBBIM-Nanomedicine, Vall d'Hebron Research Institute, Universidad Autónoma de Barcelona, Barcelona, Spain; 6 Omnia Molecular, Parc Científic de Barcelona – UB, Barcelona, Spain; 7 Networking Research Center on Bioengineering, Biomaterials and Nanomedicine (CIBER-BBN), Barcelona, Spain; University of Washington, United States of America

## Abstract

Protein components of the spliceosome are highly conserved in eukaryotes and can influence several steps of the gene expression process. RSR-2, the *Caenorhabditis elegans* ortholog of the human spliceosomal protein SRm300/SRRM2, is essential for viability, in contrast to the yeast ortholog Cwc21p. We took advantage of mutants and RNA interference (RNAi) to study *rsr-2* functions in *C. elegans*, and through genetic epistasis analysis found that *rsr-2* is within the germline sex determination pathway. Intriguingly, transcriptome analyses of *rsr-2(RNAi)* animals did not reveal appreciable splicing defects but instead a slight global decrease in transcript levels. We further investigated this effect in transcription and observed that RSR-2 colocalizes with DNA in germline nuclei and coprecipitates with chromatin, displaying a ChIP-Seq profile similar to that obtained for the RNA Polymerase II (RNAPII). Consistent with a novel transcription function we demonstrate that the recruitment of RSR-2 to chromatin is splicing-independent and that RSR-2 interacts with RNAPII and affects RNAPII phosphorylation states. Proteomic analyses identified proteins associated with RSR-2 that are involved in different gene expression steps, including RNA metabolism and transcription with PRP-8 and PRP-19 being the strongest interacting partners. PRP-8 is a core component of the spliceosome and PRP-19 is the core component of the PRP19 complex, which interacts with RNAPII and is necessary for full transcriptional activity. Taken together, our study proposes that RSR-2 is a multifunctional protein whose role in transcription influences *C. elegans* development.

## Author Summary

It is well known that splicing occurs cotranscriptionally but the functional coupling between splicing and transcription has not been studied carefully in the context of a multicellular organism in development. We took advantage of the amenable *C. elegans* genetics and genomics to demonstrate a functional relationship between RSR-2, whose yeast and human orthologs are components of the spliceosome, and transcription. Although we found that RSR-2 interacts with proteins present in the spliceosome, moderate inhibition of *rsr-2* by RNAi did not significantly affect splicing, but rather caused a decrease in transcript levels that was critical for germline sex determination. Our investigation on such a paradox of a spliceosomal component affecting transcription resulted in several lines of evidence linking RSR-2 with transcription: (i) RSR-2 immunoprecipitates chromatin resembling the ChIP-Seq profile of RNAPII, (ii) RSR-2 is present in intronless genes, (iii) *rsr-2(RNAi)* globally modifies the distribution of RNAPII along genes and its phosphorylation state, (iv) RSR-2 coimmunoprecipitates with RNAPII, and (v) RSR-2 interacts with PRP-19, which is a component of the spliceosome required for efficient transcriptional activity. Our findings raise an intriguing question: to what extent does a moderate alteration in some spliceosome components affect the gene expression process by perturbing splicing or transcription?

## Introduction

RNA splicing is a highly conserved process in eukaryotes that transforms primary transcripts, or pre-mRNAs, into mature mRNAs through the removal of intronic sequences [1], [2]. This process is accomplished by the spliceosome, which is a large and dynamic RNA-protein complex. Components of the spliceosome include five small nuclear ribonucleoprotein particles (snRNPs) and a variable number of other protein factors (over 100 have been indentified) [3]. Genetic studies of these spliceosome factors have been hampered due to their essential functions. As a consequence, most of the functional information about these proteins comes from biochemical studies to the detriment of genetic approaches. The fact that spliceosome components are well conserved through evolution enables model organisms to be used to explore the functions of individual proteins of this macromolecular complex. The manageable genetics of *C. elegans* highlight this model organism as a powerful approach to the functional dissection of the elements of this sophisticated RNA-protein machine.

SR proteins are an evolutionarily conserved family characterized by an RNA recognition motif (RRM) and a region rich in arginine and serine dipeptides (RS domain) [4]. Human SRm300/SRRM2, yeast Cwc21p, and *C. elegans* RSR-2 are called “SR-related” proteins because they do not contain RRM motifs. RS domains are important for protein-protein interactions and are present in many splicing factors. However, the RS domain can also be found in other types of proteins such as chromatin modifiers and transcriptional regulators [5]. Different gene expression steps need to be interconnected for efficient performance, and SR proteins are important in such crosstalk because they have been associated with transcription-related activities, constitutive and alternative splicing, mRNA nuclear export, nonsense-mediated decay and mRNA translation [6], [7]. An increasing number of *cis*-acting and *trans*-acting splicing mutations affecting RNA processing have been implicated in human diseases [8]. In particular, modification of SR protein sequences or alteration of their target motifs may lead to several diseases including cancer [6].

**Figure 1:**
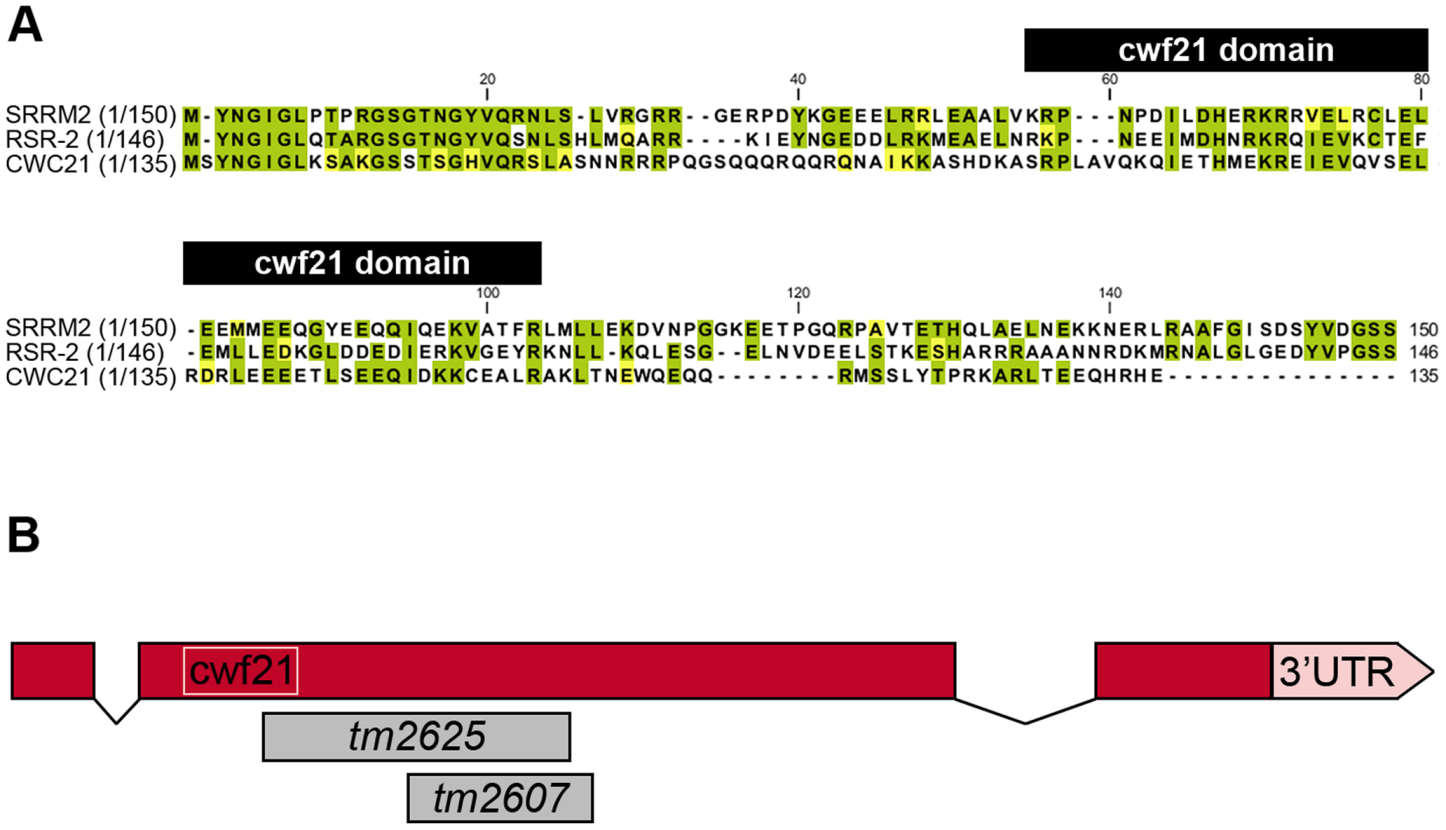
N-terminal conservation of RSR-2 and scheme of *rsr-2* gene. (A) N-terminal sequences of SRm300/SRRM2 (*H. sapiens*), RSR-2 (*C. elegans*), and the full sequence of CWC21 (*S. cerevisiae*) were compared using the ClustalW2 program. Identical and similar residues are highlighted in green and yellow, respectively. Black boxes on top of the sequences indicates the cwf21 motif. (B) Scale schematics of *rsr-2* pre-mRNA. Red boxes represent exons. Connecting lines represent introns. The white box indicates the cwf21 motif. Grey boxes represent regions deleted in *rsr-2* alleles *tm2625* and *tm2607*.

Human SRm300/SRRM2 is a much larger protein than its yeast and worm orthologs (2296 *vs.* 135 and 425 amino acids, respectively) and contains a highly conserved N-terminal region that bears a cwf21 motif. In yeast and humans, this motif is known to be required for interaction with Prp8 (Figure 1A) [9]. The 150 N-terminal amino acids of SRm300/SRRM2 and RSR-2 share 50.6% similarity and 43.3% identity. The 159 N-terminal amino acids of human SRm300/SRRM2 can functionally substitute Cwc21 in yeast, supporting the functional conservation from yeast to humans [9]. SRm300/SRRM2 was initially identified by mass spectrometry as a protein associated with SRm160 in a complex that promotes pre-mRNA splicing [10]. However, specific immunodepletion of SRm300/SRRM2 does not prevent splicing of specific pre-mRNAs in HeLa cell splicing extracts, which have previously been shown to require the SRm160/300 complex [11]. In yeast, exhaustive functional analyses have shown that, although *cwc21* mutants do not display pronounced defects in pre-mRNA processing, *cwc21* may be involved in multiple splicing-related processes by acting redundantly with other genes such as *Isy1*, *Ntc20* and *Syf2* that encode components of the Prp19-associated complex (also known as NTC complex in yeast) [9], [12]. Recently, a novel function in transcription elongation, which is probably independent of its role in splicing, has been identified for the Prp19 complex [13].

Although the SRm300/SRRM2 yeast ortholog *cwc21* is not essential for viability, the inactivation of its *C. elegans* homolog *rsr-2* by double-stranded RNA (dsRNA) microinjection produces larval-arrested animals [14]. Other specific phenotypes, including extra intestinal nuclei and synthetic genetic interactions, have been observed after inactivation of *rsr-2* through RNAi by feeding [15]. Despite the widespread observation of *rsr-2* RNAi phenotypes, a comprehensive approach to identifying *rsr-2* functional mechanisms has not so far been made [14], [15].

The fact that splicing occurs cotranscriptionally expands the possibilities of components of the spliceosome influencing transcription and/or chromatin remodeling [16]. Several lines of evidence support the influence of splicing on transcription. For example: (i) there are splicing factors that associate with the RNA Polymerase II (RNAPII) C-terminal domain (CTD) to stimulate transcriptional elongation [17]–[19], (ii) RNAPII activity is higher in intron-containing than in intronless genes [20], (iii) 5′ splice sites stimulate transcription even in the absence of splicing [21], (iv) U1 snRNPs and the Prp19 complex are present in actively transcribed intronless genes [13], [16], [22], and (v) transcriptional regulators have been identified as components of the spliceosome [23].

In this study, we describe an *rsr-2* mutation that, although informative, is disadvantageous for a genetic dissection of *rsr-2* because it induces growth arrest at early larval stages. Therefore, we have devised an *rsr-2* RNAi protocol to render a reliable and reproducible phenotype as a solid system in order to study *rsr-2* functions taking genetic and genomic approaches, including epistasis analyses, tiling arrays, chromatin immunoprecipitation-sequencing (ChIP-Seq) and RNA-Seq. We complemented these studies with proteomic analyses to demonstrate that RSR-2, despite its interaction with spliceosome components, has an impact on the transcriptional machinery that is critical to the correct development of a multicellular organism.

## Results

### Investigating *rsr-2* functions by mutations and RNAi

RNAi assays have shown that *rsr-2* is essential for *C. elegans* development. *rsr-2* RNAi approaches by microinjection or by feeding (which has a weaker effect) give rise to a variety of phenotypes, from larval and embryonic lethality to reduced brood size and sterility [14], [15], [24]. At our request, the Japanese National Bioresource Project (NBRP) generated two deletion alleles for *rsr-2* (Figure 1B). After several backcrosses to remove other possible mutations, we found that alleles *tm2607* (196 bp deletion+1 bp insertion) and *tm2625* (337 bp deletion+2 bp insertion) produced viable animals and arrested larvae respectively. The allele *tm2607* produced a truncated protein that was 65 amino acids shorter. Although several arginine and serine residues were eliminated, the truncation did not affect the cwf21 motif and the deleted region turned out not to be essential for RSR-2 functions (Figure S5B). In contrast to *tm2607*, the *tm2625* mutation is a deleterious out-of-frame deletion/insertion affecting the cwf21 motif.

**Figure 2:**
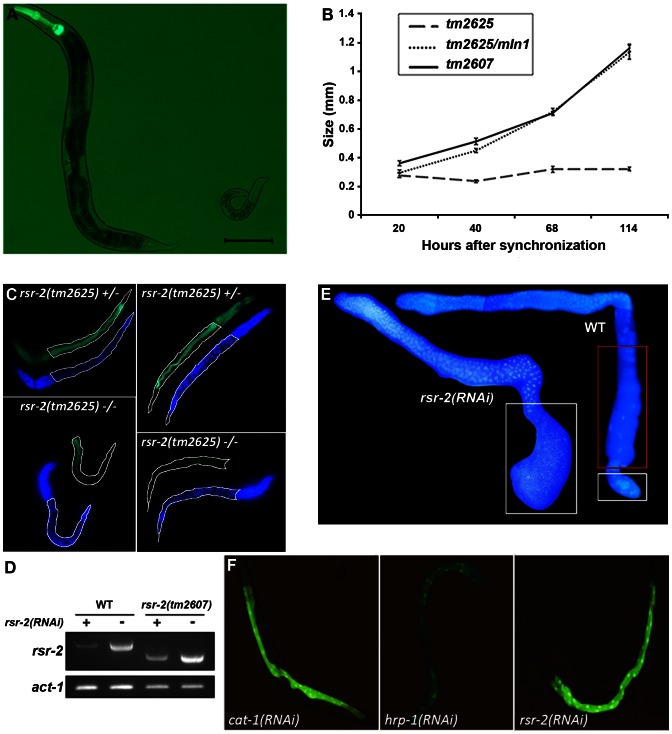
Phenotypes of *rsr-2* mutants and *rsr-2(RNAi)* animals. (A) *rsr-2(tm2625)*/*mIn1* adult (left) presents the normal size (about 1 mm) in contrast to the homozygous *rsr-2(tm2625)* worm at the same age (right). Scale bar corresponds to 0.1 mm. (B) Body sizes of *rsr-2(tm2625)* mutants, heterozygous *rsr-2(tm2625)*/*mIn1* animals and *rsr-2(tm2607)* mutants during development. Animals were grown and measured at 25°C using the NIS-Elements Software. A similar graph was obtained at 15°C (not shown). Bars indicate the standard error of the mean (SEM) for each population set. Between 6 and 13 animals were measured at each time. (C) Intestinal cells detected with MH33 antibody in heterozygous (upper panels) and homozygous (bottom panels) *tm2625* mutant larvae. Worms were counterstained with DAPI. Intestine is outlined by a white line. (D) Semiquantitative RT-PCR analysis of *rsr-2* mRNA from L4 wild type and *rsr-2(tm2607)* mutants fed with *rsr-2* RNAi (+) or *gfp* RNAi (−). *act-1* was used as endogenous control. (E) DAPI staining of dissected gonads from wild type (right) and *rsr-2(RNAi)* (left) animals. White boxes indicate the zones with sperm and the red box indicates the zone with oocytes. (F) Animals carrying a transgene consisting of introns within the GFP ORF (BL3466 strain), fed with *cat-1* RNAi negative control (left), *hrp-1* RNAi (middle) positive control and *rsr-2* RNAi (right).

We characterized *rsr-2(tm2625)* mutants and determined that the larval arrest phenotype was not temperature-dependent. After five days of post-embryonic development, animals homozygous for the *tm2625* mutation were approximately 0.3 mm long, while heterozygous *rsr-2(tm2625)* and homozygous *rsr-2(tm2607)* worms reached the standard adult size of 1 mm (Figure 2A, B). According to their size, *tm2625* animals were arrested at the L1–L2 stage [25]. We next assessed whether cell differentiation occurred correctly in these mutants by using the MH33 antibody, a marker for intestinal cell differentiation. The *rsr-2(tm2625)* animals showed a dramatic defect in MH33 staining (Figure 2C), which was in agreement with the previous observation of a dysfunctional gut detected in the progeny of animals microinjected with *rsr-2* dsRNA (Longman et al. 2001).

The acute larval arrest phenotype was difficult to track and dissect genetically. Therefore, we established an RNAi protocol to generate a population with a reproducible and milder phenotype. Synchronized L1 larvae fed at 25°C with bacteria producing *rsr-2* dsRNA showed a partial depletion of *rsr-2* mRNA levels in wild type and viable *tm2607* animals, as revealed by semiquantitative reverse transcription PCR (RT-PCR) (Figure 2D). Wild type worms exposed to *rsr-2* RNAi reached the adult stage, but these animals were sterile. Sterility was primarily caused by a defect in the sperm/oocyte switch producing worms without oocytes and with more sperm than present in wild type animals (Figure 2E). The same phenotype was observed in *rsr-2(tm2607)*; *rsr-2(RNAi)* animals (data not shown). To exclude the possibility that this *rsr-2* RNAi phenotype was caused by a general inhibition of splicing, we used the transgenic strain BL3466, which has an intron-containing GFP construct that is expressed in intestinal cells upon constitutive splicing [26]. While GFP levels were significantly reduced after RNAi-mediated knockdown of the heterogeneous nuclear ribonucleoprotein *hrp-1* positive control, *cat-1(RNAi)* negative control and *rsr-2(RNAi)* did not affect GFP expression (Figure 2F). This experiment indicates that constitutive splicing is not severely altered in *rsr-2(RNAi)* worms.

### *rsr-2* regulates the germline sex determination pathway

To determine where *rsr-2* functions within the sperm/oocyte decision pathway, we conducted epistasis experiments. We first used mutants for *gld-3* and *fog-1* that only generate oocytes and are positioned at the beginning and end of the germline sex determination pathway, respectively (Figure S1A) [27]. *gld-3(q741)*; *rsr-2(RNAi)* worms only produced sperm displaying a masculinization of germline phenotype (Mog). In contrast, *fog-1(q253)*; *rsr-2(RNAi)* worms developed only oocytes. Therefore, our RNAi assays indicated that *rsr-2* is genetically downstream of *gld-3* and upstream of *fog-1*, thereby confirming the presence of *rsr-2* in the germline sex determination pathway. We next investigated the genetic interaction of *rsr-2* with other components of the pathway (Table 1) and found that *rsr-2* is mostly upstream of *fem-3* and cooperates with *fbf-1*, *fbf-2*, *nos-3* and *puf-8* in the control of the sperm/oocyte switch.

**Table 1:** Genetic epistasis analysis of *rsr-2* in the germline sex determination pathway.

Genotype	Sp+Oo^1^ (%)	Sp^1^ only (%)	Oo^1^ only (%)	n^2^ (%)
Wild type	100	0	0	>100
Wild type, *rsr-2(RNAi)*	11	89	0	>100
*gld-3(q741)*	0	0	100	>100
*gld-3(q741)*; *rsr-2(RNAi)*	0	100	0	>100
*fog-1(q253)*	0	0	100	>100
*fog-1(q253)*; *rsr-2(RNAi)*	0	0	100	>100
*fog-2(q71)*	0	0	100	91
*fog-2(q71); rsr-2(RNAi)*	0	0	100	111
*fog-2(oz40)*	0	0	100	21
*fog-2(oz40); rsr-2(RNAi)*	0	0	100	93
*fem-3(e2006)*	0	0	100	>100
*fem-3(e2006); rsr-2(RNAi)*	16	2	82	81
*fbf-1(ok91)*	100	0	0	>100
*fbf-1(ok91); rsr-2(RNAi)*	72	28	0	72
*fbf-2(q738)*	99	0	1	>100
*fbf-2(q738); rsr-2(RNAi)*	31	50	19	121
*nos-3(q650)*	100	0	0	>100
*nos-3(q650); rsr-2(RNAi)*	72	28	0	130
*puf-8(ok302)*	93	3.5	3.5	54
*puf-8(ok302); rsr-2(RNAi)*	16	74	10	125
*puf-8(q725)*	90	10	0	56
*puf-8(q725); rsr-2(RNAi)*	25	66	9	110

Animals were grown at 25°C except *puf-8(ok302)*, which was grown at 20°C.

1 Sp: sperm. Oo: oocytes.

2 Total number of germlines scored.

The existence of genes such as *fem-3*, which regulates the germline and the somatic sex determination pathways (Figure S1B) [28], as well as the ubiquitous expression of *rsr-2* in somatic cells, encouraged us to examine whether *rsr-2* could also be involved in the somatic sex determination pathway. To address this question, we used the somatic expression of a *lacZ* reporter transgene controlled by the *fem-3* 3′UTR [29]. Whereas control animals carrying the *lacZ*::*fem-3* 3′ UTR did not express the reporter, *rsr-2* RNAi induced strong *lacZ* expression (Figure S2A). However, *rsr-2* RNAi did not cause *lacZ* expression in *lacZ*::*tra-2* 3′ UTR transgenic animals, indicating the specificity of RSR-2 in regulating 3′ UTRs (Figure S2B). Therefore, *rsr-2* acts upstream of *fem-3* in both somatic and germ cells.

During germline development, another cell fate decision takes place prior to the spermatogenesis/oogenesis switch: the transition of germ cells from a proliferating state into meiosis. If this switch fails, cells continue proliferating, resulting in a tumorous germline. To investigate whether *rsr-2* regulates this process, we first injected *rsr-2* dsRNA to induce a stronger depletion of RSR-2 during development, but *rsr-2(RNAi)* escapers that reached the adult stage were Mog sterile animals and did not have tumorous germlines. Moreover, using genetic analysis and mitotic markers, we found that *rsr-2* RNAi did not seem to affect the mitosis/meiosis transition (Figure S3). Therefore, these findings suggest that *rsr-2* is essential for some, but not all, developmental processes in the germline.

### Tiling arrays indicate that *rsr-2(RNAi)* L4 worms have slightly lower global transcript levels while splicing remains unaltered

**Figure 3:**
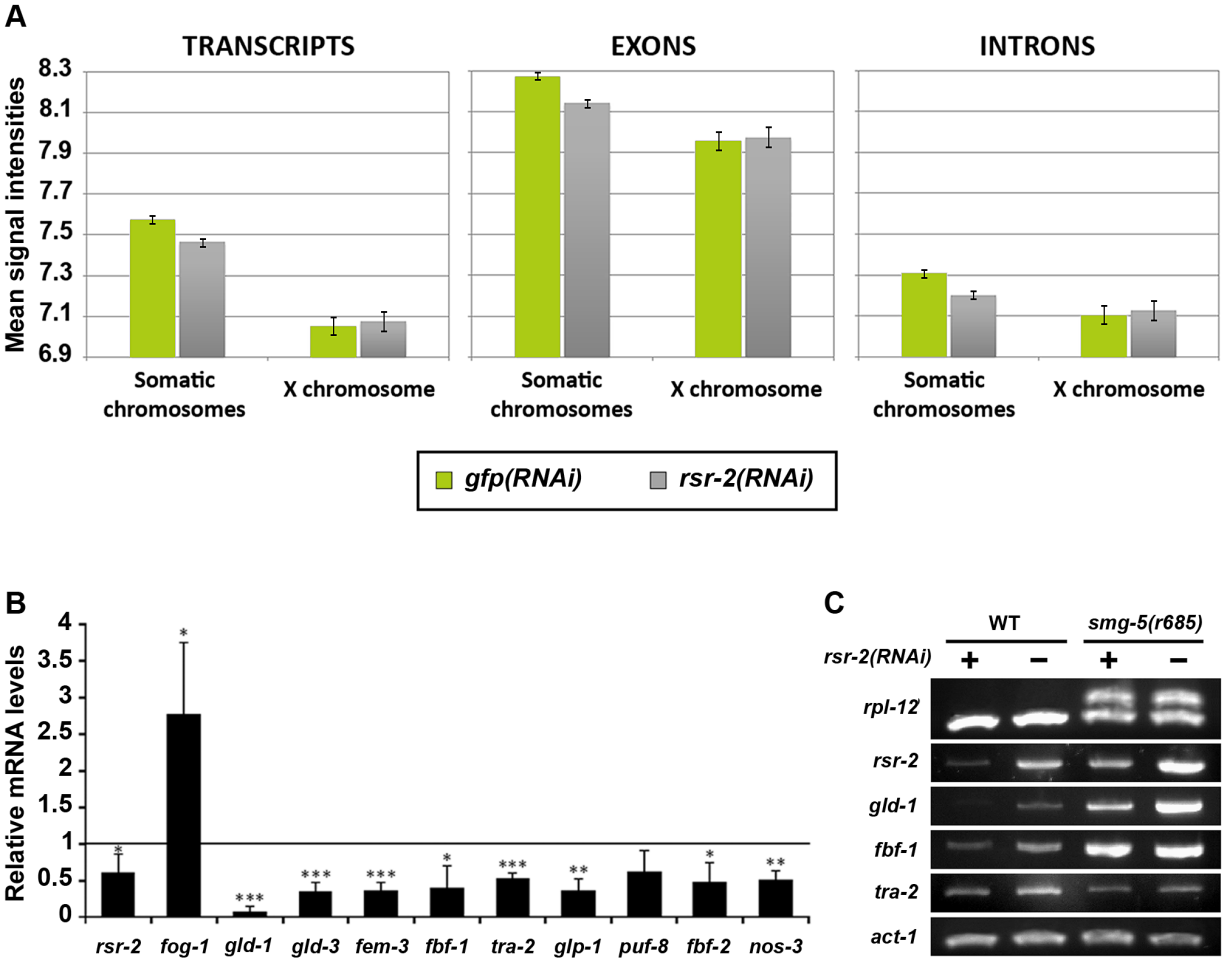
Reduction of transcript levels in *rsr-2(RNAi)* L4 animals. (A) Comparison of the average signal intensities of transcripts, exons and introns in tiling arrays between *gfp(RNAi)* and *rsr-2(RNAi)* animals. Error bars represent standard error of the mean (SEM). (B) mRNA levels of several sex determination genes upon *rsr-2* RNAi. qPCR expression data was normalized to transcript levels of *tbb-2*. mRNA levels in *rsr-2(RNAi)* animals are represented relative to the expression in *gfp(RNAi)* control animals (arbitrary value of 1.0). Three separate experiments were analyzed. Bars represent the standard deviation within each data set. Student's independent samples t-test was used to study significantly different gene expression between the two conditions: one, two and three asterisks indicate p<0.05, p<0.01 and p<0.001, respectively. (C) A subset of germline-related genes are correctly spliced upon *rsr-2* inactivation. Semiquantitative RT-PCR analysis of germline transcripts in wild type and *smg-5*(*r680*) NMD mutants treated with control *gfp* RNAi and *rsr-2* RNAi. *rpl-12* was used as a control for defective NMD pathway. *act-1* was used as an endogenous control.

To shed light on the molecular functions of *rsr-2* during *C. elegans* development, we used Affymetrix tiling arrays to examine not only transcript levels, but also intron retention events. We purified total RNA from synchronized L4 animals, when the sperm/oocyte switch takes place, grown on *gfp* and *rsr-2* dsRNA-expressing bacteria. We used two biological replicates for each condition, and raw data (in CEL files) were analyzed using the Tiling Analysis Software (TAS) developed by Affymetrix. This analysis provided information about the levels of 30,430 transcripts. To estimate gene expression in *gfp(RNAi)* and *rsr-2(RNAi)* animals, we plotted mean signal intensities for transcripts, exons and introns by chromosomes (Figure 3A). These analyses indicated that the *rsr-2* RNAi produced a slight overall reduction in transcript, exon and intron levels in all chromosomes, with the exception of the X chromosome. Mean signal intensities and normalized values for transcripts, exons and introns are shown in Table S1.

In order to obtain more information regarding the downregulated genes that cause the reduction of transcript intensity signals in *rsr-2(RNAi)* animals, we selected genes that had expression levels 20% higher or lower than the control. Using these criteria, we identified 1726 transcripts (from 1540 genes) that were upregulated and 2453 transcripts (from 2208 genes) that were downregulated. These two gene lists were uploaded to modMine (http://intermine.modencode.org), a web resource that assesses the intersection of genes of interest with gene classes generated by other studies [30]. We did not associate upregulated genes with any gene class, but we found that our list of downregulated genes was enriched in germline genes, particularly those involved in spermatogenesis (Figure S4). The decrease in transcripts required for spermatogenesis suggests that, although *rsr-2(RNAi)* animals produce an excess of sperm, these may not be properly differentiated at the developmental time when the experiment was performed. The fact that sperm-enriched genes are almost entirely absent from chromosome X [31] may explain why *rsr-2(RNAi)* worms had lower transcript levels in all chromosomes except the X chromosome.

Since our initial functional analysis of *rsr-2* was focused on germline sex determination, we performed quantitative RT-PCR (qRT-PCR) for some components of this pathway. In accordance with our tiling arrays, where we found a trend towards downregulation among germline genes, all of the tested genes with the exception of *fog-1* (enriched in sperm) [32] showed a reduction of mRNA levels in *rsr-2(RNAi)* compared with *gfp(RNAi)* control animals (Figure 3B). We next examined whether the lower level of expression of germline sex determination genes in *rsr-2(RNAi)* animals was caused by mechanisms other than splicing. Unspliced transcripts, which often contain premature termination codons (PTCs) in intron sequences, are the target of nonsense-mediated decay (NMD) degradation [33]. Consequently, if NMD is operating we would not be able to detect whether *rsr-2* RNAi causes a defect in splicing. Thus, we performed semiquantitative RT-PCR of germline transcripts from *smg-5(r860)*; *rsr-2(RNAi)* animals, in which *smg-5* mutants have a defective NMD pathway [34]. We detected abnormal transcripts in our positive control, but did not observe aberrant transcripts for any of the germline-tested genes (Figure 3C).

In conclusion, our *rsr-2* RNAi protocol partially inactivates *rsr-2*, blocking the developmental switch from spermatogenesis to oogenesis. Such *rsr-2* inactivation produces a general decrease in transcript levels that cannot be explained by altered constitutive splicing, which suggests that *rsr-2* may regulate the gene expression of processes other than splicing.

### *rsr-2* is ubiquitously expressed in somatic cells but presents a restricted pattern in the germline

**Figure 4:**
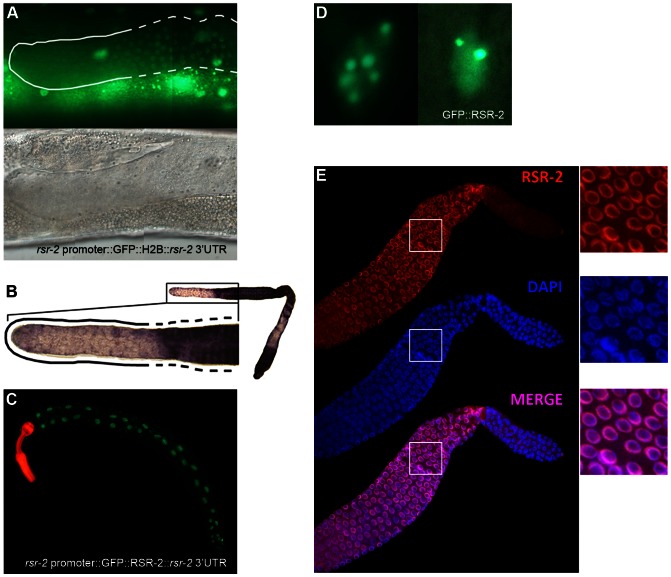
*rsr-2* expression in the soma and in the germline. (A) Expression of the transgene *cerEx01*[*rsr-2* promoter::GFP::H2B::*rsr-2* 3′ UTR] in the germline as complex array. Upper panel: detail of the distal part of the germline showing expression of GFP::H2B in the transition and meiotic zone (broken line) but not in the mitotic zone (continuous line). Bottom panel: corresponding Nomarski image. (B) Representative image of *rsr-2* mRNA in wild type germline detected by *in situ* hybridization (n = 56). Magnification of the distal part of the germline (left). The broken line labels the transition and meiotic areas whereas the continuous line marks the mitotic zone. (C) Expression of the transgene *cerEx04*[*rsr-2* promoter::GFP::*rsr-2* genomic fragment (exons, introns and 3′ UTR)]. *myo-2*::mcherry was used as a control of transformation. (D) GFP::RSR-2 forming nuclear speckles in a hypodermal cell nucleus (left) and a neuronal nucleus (right). (E) Confocal images of anti-RSR-2 immunofluorescence in the germline. Nuclei counterstained with DAPI. The three pictures on the right correspond to magnified images of the white boxes.

We next generated transgenic strains that enabled for the cellular expression of *rsr-2* and subcellular location of RSR-2 to be visualized [35], [36]. Given that extrachromosomal repetitive arrays are silenced in the germline, the transgene *rsr-2* promoter::GFP::Histone2B::*rsr-2* 3′UTR was linearized and microinjected together with digested genomic DNA to make complex arrays, which gave rise to germline expression for several generations. Since most of the gene expression regulation in the germline occurs through post-transcriptional repression [37], we included the *rsr-2* 3′UTR region in this molecular construct. This transgene showed widespread expression of *rsr-2* in somatic lineages but a restricted pattern in the germline in which the GFP signal was low or absent in the most distal part where mitosis takes place (Figure 4A). In addition, we corroborated the exclusion of *rsr-2* expression from the distal end of the germline at mRNA level by *in situ* hybridization (Figure 4B).

In order to analyze the subcellular localization of RSR-2, we generated a transgenic strain carrying the construct *cerEx04*[*rsr-2* promoter::GFP::*rsr-2* genomic fragment (exons, introns and 3′UTR)] using gene bombardment. These transgenic animals showed clear nuclear localization of RSR-2 (Figure 4C). Similar to other splicing factors [38], GFP::RSR-2 forms nuclear speckles that vary in number and size depending on the cell type (Figure 4D).

Taken together, the expression data indicate that *rsr-2* is expressed ubiquitously in somatic cells, and that RSR-2 is localized in the nucleus and concentrated in speckles. However, the lack of ubiquitous *rsr-2* expression in the germline, where different cellular processes occur at different locations, is intriguing.

### RSR-2 associates with chromatin and interacts with RNAPII affecting its distribution and phosphorylation states

**Figure 5:**
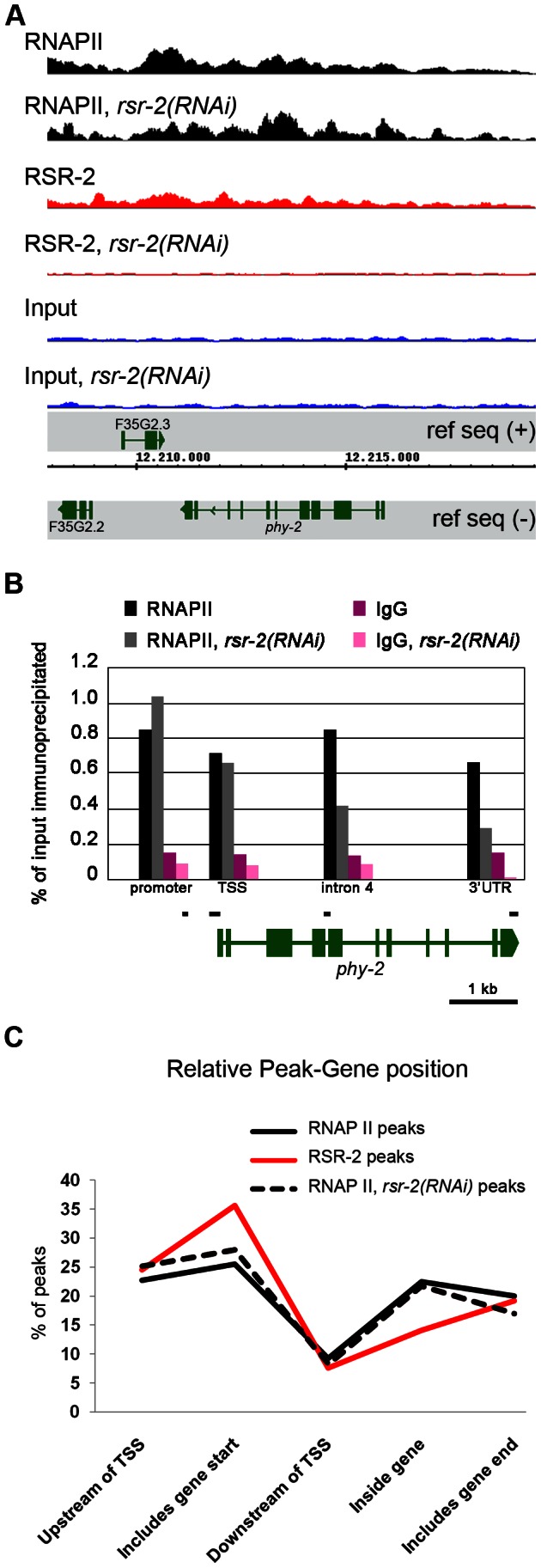
RSR-2 is associated with chromatin and modifies RNAPII distribution. (A) Snapshot of the genome browser (chromosome IV) showing ChIP-Seq data. Chromatin-binding profiles of RNAPII and RSR-2 are similar. RNAPII peaks are represented in black, RSR-2 peaks are represented in red, and input samples are represented in blue. Upon *rsr-2* RNAi, the RNAPII peak profile at *phy-2* locus shifts from the 3′ to the 5′end. RSR-2 peaks disappear upon *rsr-2* RNAi. Data was visualized with the Integrated Genome Browser (IGB) software. (B) ChIP-qPCR showing how RNAPII occupancy changes upon *rsr-2* RNAi at the *phy-2* locus. Black bars are a scaled representation of the regions covered by the primer pairs used in this experiment. (C) Distribution of RNAPII and RSR-2 ChIP peaks along five zones within an averaged gene. Statistically called peaks from a single ChIP-Seq experiment were classified with respect to their position relative to the nearest TSS. Y axis indicates the frequency of peaks within each zone. *rsr-2* RNAi slightly modifies the distribution of RNAPII along an averaged gene.

We generated a rabbit polyclonal antibody against RSR-2 (Q5092) and confirmed its specificity by western blots and in immunofluorescence assays (Figure S5A and S5C). Interestingly, anti-RSR-2 staining overlapped with chromatin as indicated by confocal images of germ nuclei (Figure 4E). We next performed ChIP-Seq experiments to investigate the association of RSR-2 with chromatin at the L4 stage using RNAPII as a control. Surprisingly, the anti-RSR-2 antibody was capable of precipitating chromatin in a ChIP-Seq pattern that resembled that of anti-RNAPII (8WG16) (Figure 5A and S10). As expected, RSR-2 peaks disappeared in worms treated with *rsr-2(RNAi)* (Figure 5A).

Given the similarities between RSR-2 and RNAPII regarding chromatin occupancy, we next wondered whether reduced levels of RSR-2 could influence RNAPII distribution and activity. As a consequence of knocking down *rsr-2* by RNAi, the shape of the RNAPII ChIP-Seq profile changed in many loci throughout the genome (Figure S10). We validated and quantified this change by ChIP-qPCR of distinct genomic fragments of the gene *phy-2*, which is downregulated in *rsr-2(RNAi)* L4 and L3 animals (Figure 5B, Table S1 and S3). Moreover, we analyzed the distribution of peaks along an averaged gene model using the SeqSolve program, employing the MACS method for peak calling [39]. This gene model was subdivided into five regions according to the peak position with respect to the closest transcription start site (TSS). The occupancy of RSR-2, compared with that of RNAPII, was greater at promoters and TSS zones and lower within genes (Figure 5C). After RNAi-mediated knock-down of *rsr-2*, RNAPII peaks were slightly enriched at the 5′ region (promoters and TSSs) and reduced at the 3′ end (peaks containing PolyA) (Figure 5C). Even after considering the noise generated from investigating different cell types in a population of L4 worms, our data suggest that, globally, *rsr-2* RNAi modified the distribution of RNAPII along the genes. This modification of RNAPII distribution may affect the transcriptional cycle and result in reduced mRNA production.

**Figure 6:**
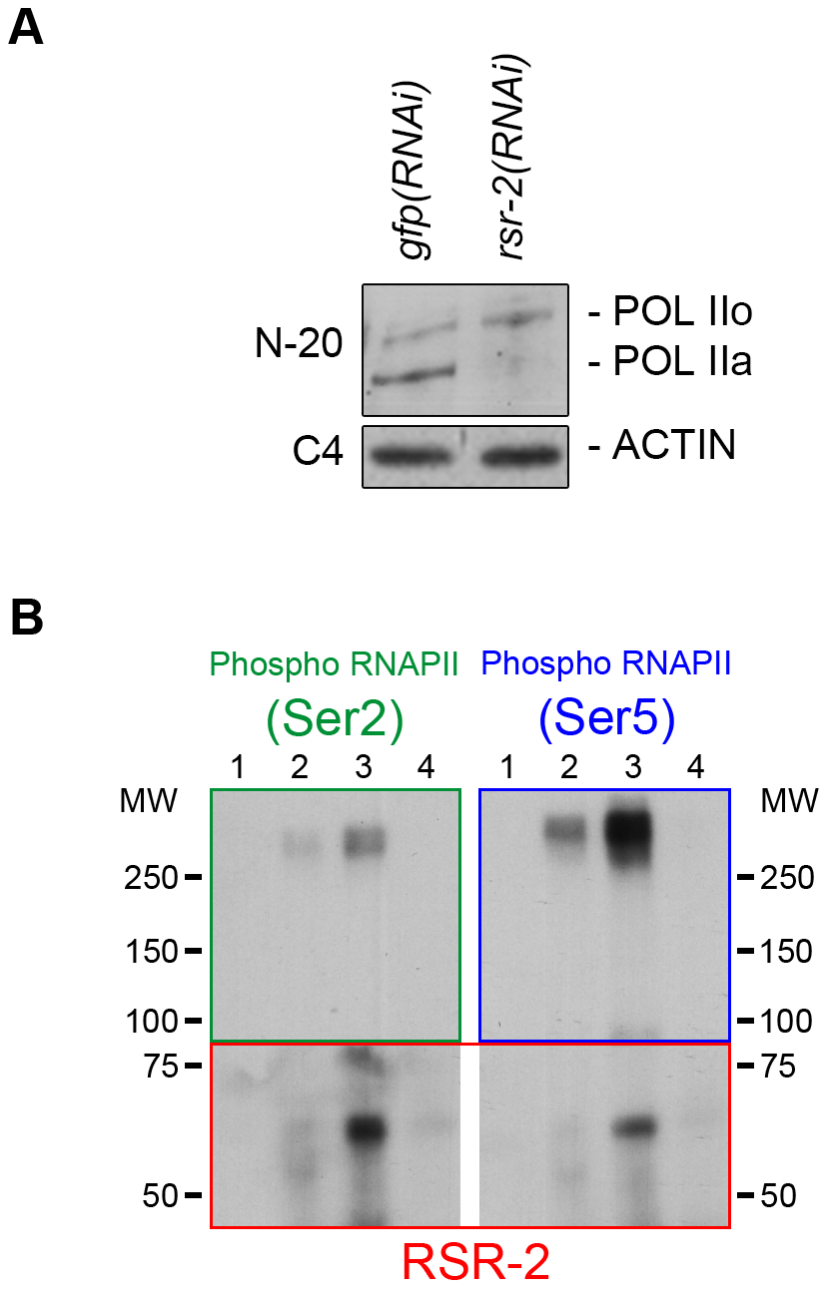
RSR-2 interacts with RNAPII and affects its phosphorylation state. RNAPII phosphoisoform detection by western blot in wild type and *rsr-2(RNAi)* worms. POL IIo is the abbreviation for the hyperphosphorylated form of the RNAPII whereas POL IIa represents the hypophosphorylated form of the RNAPII. Hyperphosphorylated RNAPII accumulates in *rsr-2(RNAi)* worms. The actin antibody C4 was used as a loading control. (A) RNAPII phosphoisoforms detected with the N-20 antibody. (B) RNAPII phosphoisoforms co-immunoprecipitate with RSR-2. IP was performed with the anti-RSR-2 antibody using an extract from a mixed-stage worm population. Eluted IP product was checked for the presence of RNAPII phosphorylated isoforms (Ser-2 and Ser-5 phosphorylation). Lane 1: Input (4% of IP), lane 2: IP with unspecific rabbit IgG antibody, lane 3: IP with RSR-2 antibody, lane 4: unspecific binding to beads. Pictures displayed are representative of a series of two experiments.

To explore further the influence of RSR-2 on RNAPII efficiency we examined whether *rsr-2* RNAi could alter the phosphorylation of RNAPII, which is informative of its activity. The C-terminal domain (CTD) of RNAPII consists of multiple repeats of a heptamer sequence that presents three serine residues (Ser-2, Ser-5 and Ser-7), which are subjected to reversible phosphorylation during transcription. In the canonical RNAPII cycle, the CTD is hypophosphorylated at the promoter and becomes phosphorylated at Ser-5 upon the initiation of transcription. As RNAPII proceeds in the 5′-to-3′ direction, phosphorylated Ser-5 (P-Ser-5) decreases and Ser-2 (P-Ser-2) increases, favoring the recruitment of 3′-end processing factors [40]. We used the anti-RNAPII antibody 8WG16, which binds to both hyperphosphorylated and hypophosphorylated isoforms [41], [42], and found that *rsr-2(RNAi)* extracts have more hyperphosphorylated RNAPII than control *gfp(RNAi)* extracts as assayed by western blot (Figure S6). We validated this observation by using a different RNAPII antibody (N-20), which also recognizes the two phosphorylation states (Figure 6A). This result could be contradictory since *rsr-2* RNAi produces an increment of active RNAPII and, at the same time, a slight global decrease in the number of transcripts. However, as argued in the discussion, an increment of active RNAPII does not always correlate with more efficient transcription.

Finally, we investigated whether RSR-2 and RNAPII can interact by using a coimmunoprecipitation assay (CoIP). We performed CoIP and found that both Ser-2 and Ser-5 RNAPII phosphorylated forms are able to coimmunoprecipitate with RSR-2. It seems that the interaction between RSR-2 and P-Ser-5 has a stronger binding affinity than that between RSR-2 and P-Ser-2 (Figure 6B), which supports the observed biased abundance of RSR-2 towards the 5′ end of genes. Together, these observations suggest that RSR-2 may be involved in the regulation of transcription based on the findings that (i) RSR-2 physically interacts with RNAPII, (ii) RSR-2 associates with chromatin with a pattern similar to that of RNAPII, and (iii) the reduction of RSR-2 levels modifies RNAPII distribution and the balance of RNAPII phosphorylated forms.

### Splicing-independent recruitment of RSR-2 to transcriptionally active genes

**Figure 7:**
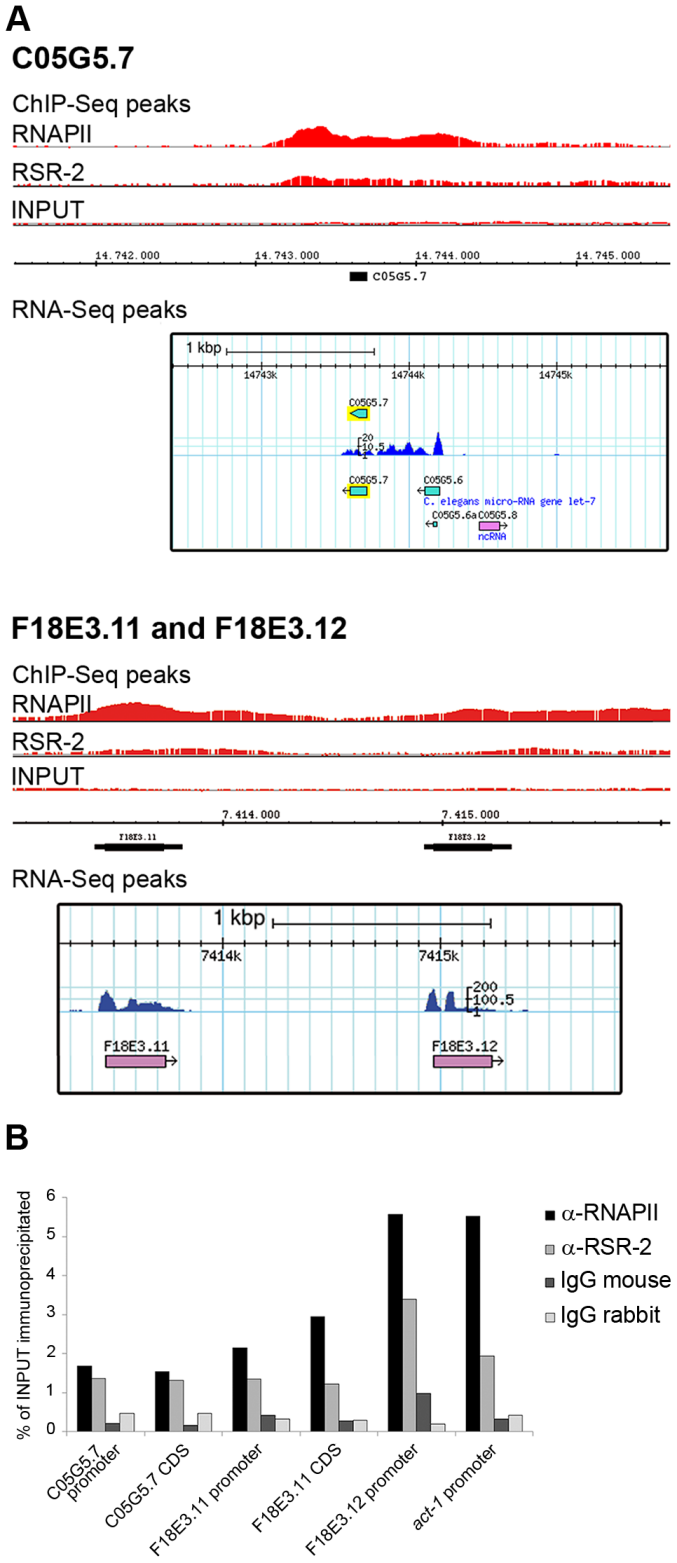
RSR-2 binds to intronless genes. (A) Chromatin-binding profiles of RNAPII and RSR-2, and RNA-Seq reads in intronless genes. RNA-Seq reads correspond to the N2 mid-L4 stage dataset from the modENCODE consortium [82]. (B) ChIP-qPCR for intronless genes with mouse anti-RNAPII (8WG16), rabbit anti-RSR-2 (Q5092) and unspecific mouse and rabbit IgG antibodies (sc-2025 and sc-2027 respectively). All qPCR values are represented as the percentage of input immunoprecipitated.

Our ChIP-Seq analyses identified 6889 RNAPII peaks in 4679 genes, and 5445 RSR-2 peaks in 4338 genes (p-value cutoff 0.00001) (Table S2). Most of these peaks were present in both RSR-2 and RNAPII ChIP-Seq profiles (Figure S7). Interestingly, 74 of the RSR-2 peaks were located in intronless genes (551 intronless genes identified in the WS220 version of the genome). This finding indicates that the recruitment of RSR-2 to chromatin can be independent of splicing. Since the *C. elegans* genome is quite compact, we scanned the Genome Browser for RSR-2 peaks located at single exon genes that did not have genes expressing multi-exonic transcripts in the immediate vicinity (Figure 7A). To further validate these peaks we performed ChIP-qPCR along several intronless genes and concluded that both proteins, RNAPII and RSR-2, bind to intronless genes at regulatory and coding sequences (Figure 7B).

### RSR-2 interacts with proteins related to splicing and transcription

We next immunopurified RSR-2-containing complexes from two biological replicates of wild type mixed-stage worm populations. Most of the proteins identified as RSR-2 interactors were components of the spliceosome, but we also detected proteins related to stress response, transcription, chromatin regulation, translation and ubiquitination (Table 2 and S4). A large number of peptides of the core spliceosome protein PRP-8 were identified, which is consistent with the physical interaction found between Cwc21p and Prp8p in yeast [9], [43]. Moreover, we detected interactions with snRNPs, which have also been observed in its human counterpart SRm300/SRRM2 [44]. Interestingly, we identified three proteins that are functionally related to both splicing and transcription: SKP-1, KIN-3, and PRP-19. SKP-1 is the ortholog of PRP45 in yeast and SKIP/SWN1 in humans. It has previously been shown that SKIP/SWN1 plays independent roles in splicing and transcription elongation [45]. KIN-3 encodes the ortholog of the human catalytic subunit of CKIIα, which is a protein kinase that phosphorylates several substrates regulating transcription and physically interacts with the splicing factor PRPF3 [46]. Finally, PRP-19 is the ortholog of human PRPF19, and the conserved Prp19 complex (Prp19C) is involved in pre-mRNA processing and transcriptional efficiency [13].

**Table 2:** Protein members of RSR-2-containing complexes.

Description	α-RSR-2 IP1	α-RSR-2 IP2
	(#peptides)	(#peptides)
**RNA metabolism**		
Pre-mRNA-splicing factor 8 homolog (*prp-8*)	12	2
Small nuclear ribonucleoprotein Sm D3 (*snr-1*)	1	2
Probable U2 small nuclear ribonucleoprotein A′ (*mog-2*)	2	
Probable small nuclear ribonucleoprotein Sm D1 (*snr-3*)	1	1
Probable small nuclear ribonucleoprotein E (*snr-6*)	1	1
Pre-mRNA-splicing factor CWC22 homolog (*let-858*)	1	1
Probable small nuclear ribonucleoprotein-associated protein B (*snr-2*)	1	
rRNA 2′-O-methyltransferase fibrillarin (*fib-1*)		1
Nucleolar complex protein 2 homolog (*pro-2*)	1	
**RNA metabolism and transcription**		
Pre-mRNA-processing factor 19 homolog (*prp-19)*	6	4
Transcriptional cofactor T27F2.1 (*skp-1*)	1	2
Casein kinase II subunit alpha (*kin-3*)	9	1
**Stress response**		
Stress-induced protein 1 (*sip-1*)	7	4
Heat shock 70 kDa protein A (*hsp-1*)	2	1
**Ubiquitination**		
E3 ubiquitin-protein ligase *siah-1* (*siah-1*)	2	2
Polyubiquitin-A (*ubq-1*)	3	
**Chromatin**		
Histone H2A (*his-3*)	1	
Lysine-specific demethylase *rbr-2* (*rbr-2*)		1

Some of the proteins identified from mass spectra of peptides recovered from complexes immunopurified with an antibody against RSR-2. Two biological replicates are immunoprecipitated (IP). After the immunopurification, the peptide mixture is analyzed by LC/MS coupled to nano-HPLC. The total number of unique peptides identified for each protein is indicated in the columns under the antibody heading. All of unique peptides have been identified in the immunoprecipitated sample but not in the control sample immunopurified with an unspecific rabbit antibody against IgG.

**Figure 8:**
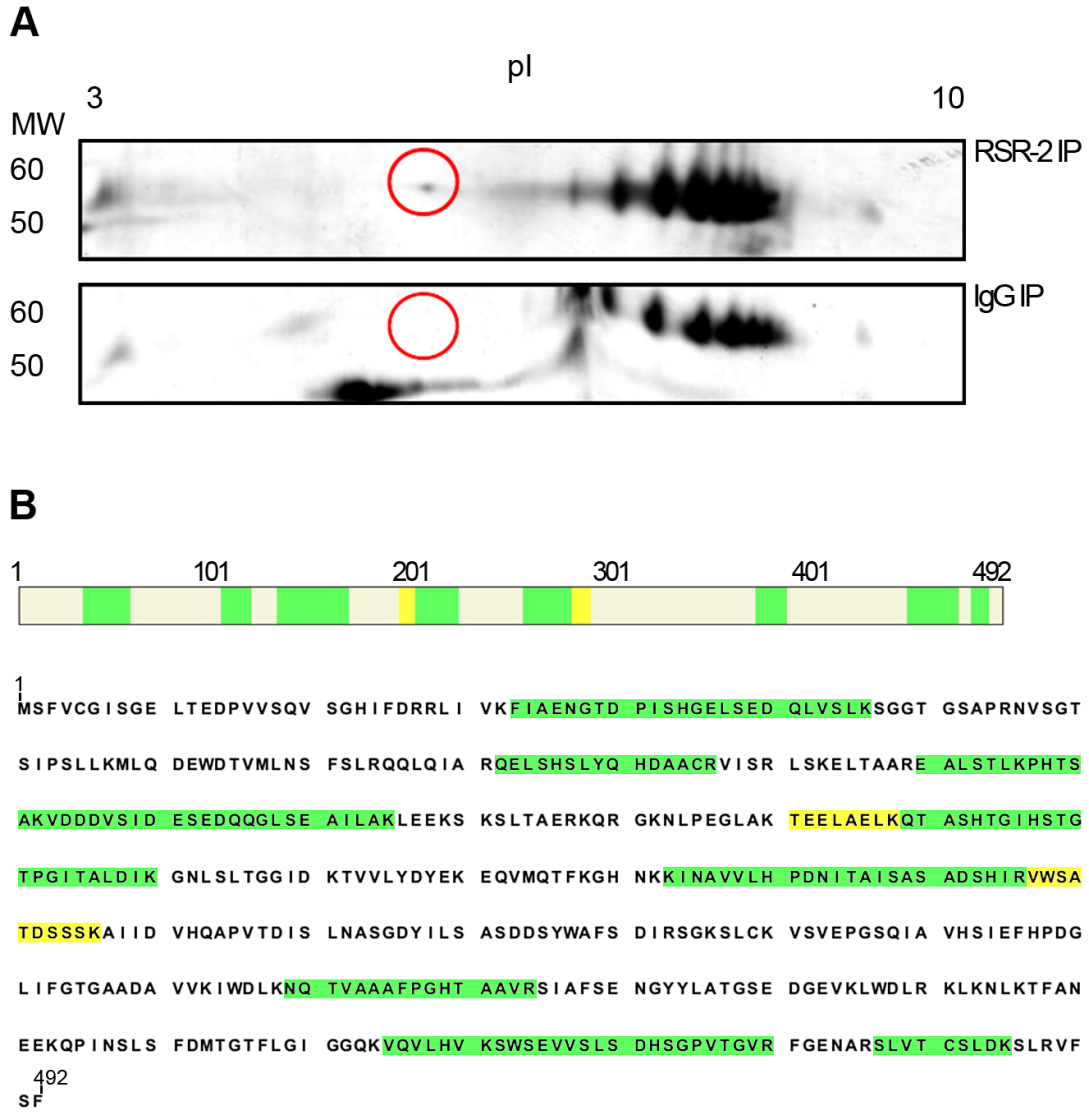
RSR-2 interacts with PRP-19. (A) After immunopurifying protein complexes from *C. elegans* mixed staged populations with a rabbit RSR-2 specific antibody and an unspecific rabbit antibody against IgG, both protein samples were run in bidimensional gels. Gels were silver-stained and the spot of interest was manually excised for further LC-MS/MS analysis. Proteins were separated in the bidimensional gel by molecular weight (MW) and isoelectric point (pI). The selected differential spot is circled in red. (B) This spot corresponds to the pre-mRNA processing factor PRP-19. 13 unique peptides with a PSM (Peptide Spectral Match) of 22 match the PRP-19 sequence. High confidence identity peptides are represented in green (q<0.01) and medium confidence identity peptides are represented in yellow (q<0.05).

We expanded our proteomic analysis by performing two-dimensional electrophoresis (2-DE) and mass spectrometry analysis of one differential spot between a sample immunoprecipitated with anti-RSR-2 and a control sample (non-specific rabbit immunoglobulin) (Figure 8). Interestingly, we found 22 unique peptides corresponding to PRP-19, which validated its interaction with RSR-2. Thus, our proteomic analyses of RSR-2-containing complexes support the hypothesis that RSR-2 is part of the spliceosome but has the capacity to influence the transcriptional machinery through several protein-protein interactions.

### Convergences and divergences between *rsr-2* and *prp-8*

**Figure 9:**
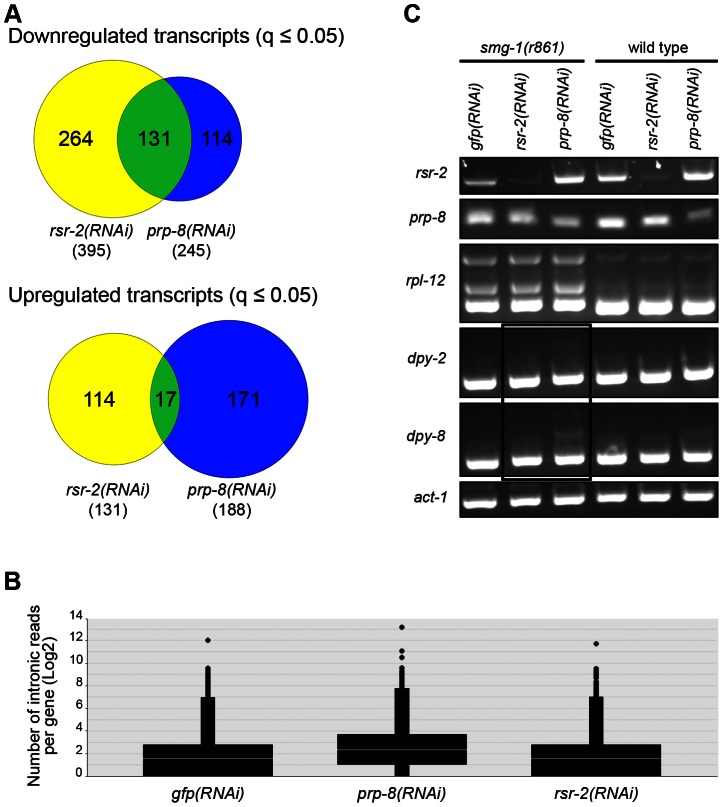
RNA-Seq of *rsr-2(RNAi)* and *prp-8(RNAi)* L3 animals. (A) Venn diagrams of down- and up-regulated transcripts in *rsr-2(RNAi)* (yellow) and *prp-8(RNAi)* (blue) L3 animal populations. Overlapping transcripts between the two samples are represented in green. (B) Graph representing the abundance of RNA-Seq reads mapped in intronic regions without internal genes corresponding to control *gfp(RNAi)*, *prp-8(RNAi)*, and *rsr-2(RNAi)* animals. (C) Semiquantitative RT-PCR of wild type and *smg-1(r861)* worms treated with *gfp* RNAi control, *rsr-2* RNAi and *prp-8* RNAi. *rpl-12* was used as a positive control for intron retention in NMD-defective animals. *act-1* was used as an endogenous control. The black box indicates the accumulation of aberrant mRNAs for *dpy-2* and *dpy-8* in the *smg-1(r861)*; *prp-8(RNAi)* sample, but not in the *smg-1(r861)*; *rsr-2(RNAi)* sample.

Since PRP-8 interacts with RSR-2 and is a protein located at the catalytic core of the spliceosome, we were interested in comparing the transcriptomes of *rsr-2(RNAi)*, *prp-8(RNAi)* and control *gfp(RNAi)*. We conducted RNA-Seq analysis in L3 animals because *prp-8(RNAi)* animals exhibited a severe developmental delay after L3 in our RNAi feeding conditions. Interestingly, there was a substantial number of transcripts (much more than expected by chance, 131 *vs.* 7 respectively) that were significantly downregulated (q≤0.05) in both *rsr-2* and *prp-8* deficient animals (Figure 9A) (Table S3). However, the overlap among upregulated genes was meaningless (Figure 9A). These results suggest common functions for RSR-2 and PRP-8 in controlling gene expression. Therefore, we further analyzed the common list of transcripts downregulated in *prp-8* and *rsr-2(RNAi)* animals using modMine [30]. We found gene ontology (GO) terms for “body morphogenesis” and “molting cycle”, which included genes with high expression levels during postembryonic development.

Sequencing analyses revealed that *prp-8(RNAi)* transcriptomes accumulated more reads in introns than *rsr-2* and *gfp(RNAi)* samples (Figure 9B). Therefore, using semiquantitative RT-PCR, we investigated the retention of introns, in wild type N2 animals and non-mediated decay (NMD) mutants, for two of the genes downregulated in both *rsr-2* and *prp-8(RNAi)* animals (Figure 9C). We observed intron retention events in *prp-8(RNAi)* but not in *rsr-2(RNAi)* samples. Thus, we concluded that although both PRP-8 and RSR-2 are part of the spliceosome, after partial inactivation through RNAi the contribution to splicing catalysis was only obvious for PRP-8. Nevertheless, we cannot rule out the possibility that the full depletion of RSR-2 activity could somehow affect pre-mRNA processing.

We next assessed the possible effect of *rsr-2* RNAi in alternative splicing by evaluating the predominance of spliced forms from our RNA-Seq data. We studied ten genes that have been reported to present alternative splicing at the L3 stage [47] and did not observe drastic variation in the balance of these alternative spliced transcripts (Figure S9). However, we cannot rule out the possibility that a stronger depletion of RSR-2 levels could favor one particular transcript that influences worm development.

Taken together, we hypothesize that during development, mild *rsr-2* and *prp-8* deficiencies may primarily affect highly transcribed genes. Nevertheless, the involvement of defects in splicing or in transcription in the eventual reduction of gene expression requires further exploration.

## Discussion

In recent decades, our functional knowledge of spliceosome components has largely been driven by biochemical studies, because genetic analyses have been hampered by the essential functions of these proteins. The discovery of RNAi and the possibility of modulating its efficacy allowed for the mimicking of phenotypes caused by hypomorphic alleles. This study shows that reduced levels of *C. elegans* RSR-2 blocks the switch from spermatogenesis to oogenesis. Since RSR-2 is a homolog of the human splicing coactivator SRm300/SRRM2 and the yeast *cwc21* spliceosome component, it was expected that *rsr-2* inactivation would primarily affect the splicing process. However, we did not find appreciable alterations in constitutive splicing in animals with reduced levels of RSR-2. Instead, we observed a slight global reduction in the abundance of transcripts at L4 when the sperm/oocyte switch takes place. To explain the *rsr-2(RNAi)* phenotype in germline sex determination we propose a model in which low RSR-2 levels cause a decrease in the expression of genes, both activators and repressors, within the complex gene network governing the sperm/oocyte switch. The outcome of such global downregulation is that the abundance of FEM-3 protein is not low enough to trigger the switch to oogenesis (Figure S8). Grades of *rsr-2* inactivation and the consequent reduction of the transcriptional levels required to manifest a phenotype may vary depending on each developmental process. Our study suggests that an RSR-2 deficit may more strongly affect processes driven by the action of high levels of transcripts at certain developmental times, such as during germline sex determination at the L4 stage or cuticle formation at the L3 stage. A parallelism could be established with the effect of hypomorphic mutations of certain splicing factors that cause the rare autosomal dominant human disease Retinitis Pigmentosa (adRP). adRP patients suffer a pathology of the retina, which is a tissue with a high transcriptional activity in adults, whereas other tissues are unaffected.

Interestingly, *mog* genes are a group of splicing-related genes that are also required for the sperm-to-oocyte switch [2], [48]–[52]. There are similarities and differences between *mog* genes and *rsr-2*, the former of which includes: (i) RSR-2 and MOG proteins are nuclear, (ii) *rsr-2* and *mog* are required for somatic repression of a *lacZ*::*fem-3* 3′UTR transgene [29], (iii) *mog* genes and *rsr-2* are located upstream of *fem-3* in the sex determination pathway [27], (iv) *mog* mutants, similar to *rsr-2(RNAi)* animals, do not show general splicing defects [50], [52]–[54], (v) we have detected a physical interaction between MOG-2 and RSR-2 (Table 2) and (vi) we have shown a link between RSR-2 and transcription, and *mog* genes interact with the zinc-finger protein MEP-1 [27], [55]. However there are differences that set *rsr-2* apart from *mog* genes, including: (i) most of the MOG proteins are DEAD/H-box RNA helicases, whereas RSR-2 lacks motifs with the capacity to bind or rearrange RNA, (ii) in contrast to *rsr-2*, all *mog* genes have a role in germline proliferation and function synthetically with *gld-3* [48], (iii) a restricted expression pattern in the germline is unique for *rsr-2*, and (iv) *rsr-2(RNAi)* at 20°C in the *rrf-1(pk1417)* background, which confers RNAi resistance in the soma, produced 40% of Mog animals (data not shown) while RNAi of *mog* and other splicing-related genes produce much lower proportion of Mog worms under the same conditions [56]. Therefore, the similarities and differences between *rsr-2* and *mog* genes suggest that they may share some functions but also have independent roles.

RNA processing can occur before transcription is completed, so both molecular mechanisms need to be functionally coupled to ensure efficient gene expression [57], [58]. Proteomic analyses of complexes containing RSR-2 and its orthologs have identified not only pure splicing factors, but also proteins involved in chromatin remodeling and transcription [9], [12], [44], [59]. Due to the recently identified role of the PRP19 complex in transcriptional elongation, the physical interaction of RSR-2 and its yeast and human orthologs with components of PRP19 complexes [9], [12], [44], [60] are particularly helpful for explaining the participation of RSR-2 in transcription proposed in this study [13]. Such interactions would also be functional because the yeast PRP19 complex components Isy1 and Cwc21 are functionally redundant [9], [43]. Moreover, the PRP19 complex, similar to RSR-2, is recruited to intron-containing as well as intronless genes in a transcription-dependent manner [13]. In addition, the physical interaction between RSR-2 and the worm orthologs of human CKIIα and SKIP/SWN1, which are implicated in transcriptional elongation [18], [45], [61], further reinforces the nexus between the RSR-2 interactome and regulation of transcriptional efficiency.

Beyond these interactions of RSR-2 with proteins playing roles in transcription, our study reveals that RSR-2 coimmunoprecipitates with RNAPII. The interaction between RSR-2 and RNAPII is not rare since some splicing factors directly interact with the C-terminal tail (CTD) of the polymerase [62]. Moreover, reduced levels of RSR-2 have an impact on global RNAPII distribution and phosphorylation. The increase of the active hyperphosphorylated RNAPII form observed after *rsr-2* RNAi does not necessarily result in more efficient transcriptional activity. During animal development RNAPII often stalls or pauses just after the initiation of transcription because the accumulation of transcriptionally engaged RNAPII close to the TSS is a strategy for regulating the expression of genes in stimulus-responsive pathways and of regulators in signaling pathways [41], [63], [64]. Moreover, induced DNA damage causes CTD hyperphosphorylation beyond normal levels, and this hyperphosphorylation is associated with slow transcription [65].

This study has shown that RSR-2 is committed to the two largest nuclear gene expression machineries, its subcellular location being compatible with this. RSR-2 distribution is mainly nuclear: it is present in nuclear speckles or clearly overlaps with DNA as in the case of the germ cells. Human SRm300/SRRM2 is also nuclear and localizes to nuclear speckles in a range of human cells types [11], [66], [67]. Although several RNA processing factors have been detected in speckles of this type, transcriptional factors are also detected within such structures [38]. In fact, although nuclear speckles are commonly known as RNA processing bodies, they are often in the close vicinity of transcriptionally active chromosome territories [68].

Although this report is more concerned with the evidence for a new role of RSR-2 in transcription, we cannot rule out the possibility that the total absence of RSR-2 might somehow affect pre-mRNA processing. We also cannot discount the possibility that RSR-2 could play a potential role in alternative splicing, since transcriptional elongation can affect the outcome of alternative splicing events [69]. However, *rsr-2(RNAi)* animals did not show any evident alteration in the expression of alternative splice forms.

Some of our results relate *rsr-2* with *prp-8*, which is a gene encoding a protein located at the catalytic core of the spliceosome. First, our proteomic studies revealed an interaction between RSR-2 and PRP-8. Second, using permissive RNAi conditions (feeding from L2–L3 stage) to inactivate *prp-8* and F53B7.3 (Isy1 yeast homolog) genes, we observed the Mog phenotype (data not shown). Third, RNA-Seq revealed that PRP-8 and RSR-2 have target genes in common, which are downregulated in *prp-8* and *rsr-2(RNAi)* animals. However, an excess of intron retention was only observed in *prp-8(RNAi)* worms. There are two hypotheses to explain why *prp-8* and *rsr-2* regulate the expression of a similar subset of genes. First, *rsr-2* RNAi may affect splicing by producing immature transcripts that are undetectable in our experimental conditions (because RNA-Seq coverage is insufficient or semiquantitative RT-PCR is unable to detect low levels of immature transcripts). The second possibility is that partial *prp-8* inactivation may cause a defect in transcription as a consequence of the functional coupling between the splicing and transcriptional machineries. In other words, a defect in *prp-8* function might compromise splicing efficacy and transcription may respond to such a drawback by downregulating its activity. Thus, slowing down the active RNAPII would provide sufficient time for the spliceosome to rectify the problem and avoid the accumulation of immature transcripts.

Many splicing factors are essential genes that do not have functional redundancies that can cover their loss of function. It is possible that, as demonstrated in cells, during animal development splicing presents a buffering capacity due to the coupling with transcription to deal with partial loss of function of some spliceosome components.

Our data fit a model in which RSR-2 is a component of both the spliceosome and the RNAPII complex, but where a partial reduction of RSR-2 levels has a greater impact on transcriptional efficiency than on pre-mRNA processing. Importantly, the effect on transcription seems to be critical for *C. elegans* development and viability. The use of nuclear run-on assays and ChIP experiments with antibodies specific to various RNAPII forms in *C. elegans* will aid our understanding of how RSR-2 regulates transcription of genes [70], [71]. Since *rsr-2* was identified as a genetic interactor of *lin-35* Retinoblastoma [15], another aspect that needs to be explored is the impact of *rsr-2* inactivation on chromosome modifications and nucleosome positioning due to the functional connection between chromatin, splicing and transcription [72].

## Materials and Methods

### Strains

Standard methods were used to culture and manipulate worms [73]. We used the wild type strain Bristol N2 and the following alleles and balancers: *fog-1(q253) I*, *smg-5(r860) I*, *smg-1(r861) I*, *rrf-1(pk1417) I*; *glp-1(oz264) III*, *rrf-1(pk1417) I*; *gld-3(q730) II*, *rsr-2(tm2625)/mIn1[(dpy-10(e128)mIs14(myo-2::GFP)] II*, *rsr-2(tm2607) II*, *gld-3(q741)/mIn1[dpy-10(e128)mIs14(myo-2::GFP)] I*, *fbf-1(ok91) II*, *fbf-2(q738) II*, *nos-3(q650) II*, *puf-8(ok302) II*, *puf-8(q725) II*, *pha-1(e2123) III*, *fem-3(e2006) IV*, *and fog-2(q71) V*. We also used the following transgenes: *qIs43[lacZ::fem-3(+)], qIs15[lacZ::fem-3(q96gf)], wt8Di[lacZ::tra-2(+)], inIs173[pNvitgfp], cerEx01[rsr-2 promoter::gfp::Histone2B::rsr-2 3′UTR]*, and *cerEx04[rsr-2 promoter::gfp::rsr-2 genomic fragment+rsr-2 3′UTR]*.

### RNA-mediated interference (RNAi)

To induce RNA-mediated interference (RNAi) by feeding, nematode growth medium (NGM) plates were supplemented with 50 µg/ml ampicillin, 12.5 µg/ml tetracycline, and 1 mM IPTG. Plates seeded with the corresponding RNAi clone, validated by PCR and/or sequencing, from either ORFeome library [24] or Ahringer library [43] were used to feed wild type synchronized L1 animals. To interfere *rsr-2* expression by microinjection, dsRNA was synthesized by using MEGAscript T7 kit (Ambion Cat. No. AM1333). Wild type young adults were injected with 1 ng/µl of *rsr-2* dsRNA and F_1_ progeny was grown at 25°C.

### Semiquantitative RT-PCR

Synchronized L4 worms were harvested [25], washed and frozen in TRIReagent (MRC Inc, Cat. No. TR-118) to perform total RNA isolation. cDNA was synthesized with oligo(dT) primers using the RevertAid H Minus First Strand cDNA synthesis kit (Fermentas Cat.No. K1632) following the manufacturer's instructions.

### *In situ* hybridization of mRNA

*In situ* hybridization was performed mainly following the protocol described by Lee & Schedl [74], although gonad dissection, washes and incubations were performed on a multi-well Pyrex plate (Electron Microscopy Sciences). A sense probe was used as a negative control.

### Immunostaining

Dissected gonads were fixed with 4% paraformaldehyde (Electron Microscopy Sciences Cat. No. 15710) and processed following standard immunostaining protocols. We used the freezing-cracking protocol for larval immunostaining [75]. The RSR-2 antibody was raised in rabbits against the immunogen sequence of the RSR-2 protein, which comprises aminoacids 39 to 138, and was affinity purified (Sdix, Strategic Diagnostics Inc. USA). Antibodies and dilutions used for immunofluorescence in this study were: anti-RSR-2 (Q56092) 1∶600; anti-GFP (A11120) (Molecular Probes) 1∶100; anti-intermediate filament (MH33) (DSHB) 1∶40; Alexa Fluor 488 IgG (Molecular Probes) 1∶400; and Alexa Fluor 568 IgG (Molecular Probes) 1∶400.

### Generation of GFP reporters and transgenic animals

Molecular constructs were made by the Gateway recombinational cloning system (Invitrogen). Transgenic animals were generated by microinjection or bombardment with gold particles (Biolistic Helium Gun, Caenotec). Bombardment: N2 young adults were shot with 16 ng/µl of the reporter *cerEx04*. Transgenic animals from the progeny were selected through the puromycin/neomycin selection system [76]. Microinjection: 4 ng/µl of the linearized molecular construct *cerEx01* were microinjected together with digested bacterial genomic DNA (80 ng/µl) and linearized pRF4 (2 ng/µl) (roller marker) to make complex arrays.

### LacZ reporter assay

Animals carrying different *lacZ::*3′UTR reporter transgenes were synchronized at the L1 stage and fed with RNAi clones at 25°C. Adults were heat shocked for 2 hours at 30°C, recovered for 2 hours at 25°C, and processed to score for *lacZ* expression. Processing of samples and scoring was performed as described by Gallegos et al. [29].

### Tiling arrays

Wild type N2 hermaphrodites were synchronized at the L1 stage [25] and total RNA from *rsr-2* and *gfp(RNAi)* worms grown at 25°C was isolated 36 hours after L1 arrest. cDNA was synthesized and hybridized to Affymetrix *C. elegans* tiling array 1.0 (ref 900935). Affymetrix Tiling Analysis Software (TAS) V.1.1.02 was used to analyze the data. Briefly, the array signals were extracted comparing the mismatch probe values (MM) with their perfect match probes (PM). Then, the biological replicates were quantile-normalized and smoothed by a sliding window procedure (window size of 110 bp), and signals were scaled to a median intensity of 100. To detect regions of active transcription, interval analyses were made to obtain the corresponding transfrags, using in the algorithm a maxgap of 30 bp and a minrun of 50 bp for all data sets. Finally, using the tools implemented in the Galaxy platform [77], [78], and based on the information contained in the worm genome assembly WS180, the array signal values were used to infer the mean signal intensity for each gene in control and *rsr-2(RNAi)* worms, as an indirect estimate of whole gene expression. These estimates were used to recognize up- and down-regulated genes in the knocked-down population, and also to characterize the transcription patterns of each chromosome.

### Real-time PCR assays

For RNA analysis, total RNA was isolated and cDNA synthesized with oligo(dT) primers. For DNA analysis, chromatin was immunoprecipitated and purified as detailed later in this section. In both cases, a LightCycler 480 SYBR Green I Master kit (Roche Cat. No. 04707516001) was used to determine gene expression or the amount of immunoprecipitated DNA respectively.

### Protein analysis

Western blots were performed according to standard protocols (the detailed protocol can be found in the supporting Text S1). Antibodies and dilutions used were: anti-RSR-2 Q5092 (Sdix) (1∶500), anti-RNAPII N-20 (Santa Cruz Biotech) (1∶1000), phospho Ser-2 RNAPII A300-654A (Bethyl Laboratories, Inc) (1∶5000), phospho Ser-5 RNAPII A300-655A (Bethyl Laboratories, Inc) (1∶5000) and anti-ACTIN C4 (MP Biomedicals) (1∶500).

### Chromatin immunoprecipitation - sequencing (ChIP-Seq)

ChIP assays were carried out as described previously by Zhong et al., with minor modifications [42]. Worms were collected at the L4 stage and 0.5 ml of packed larvae (≈2.2 mg of total protein) were crosslinked, sonicated and immunoprecipitated using anti-RSR-2 (Q5092) and anti-RNAPII (8WG16) antibodies (10 µg of each antibody). The immunoprecipitated DNA fragments and the input DNA were used to prepare libraries for sequencing using the Illumina GA platform, through the modENCODE sequencing facility set up in the Snyder laboratory (Stanford University). In order to run four samples in one flow cell, sequencing libraries were bar-coded and multiplexed as described in Lefrançois et al. [79]. ChIP-Seq fastq files were processed in Galaxy and mapped against the *C. elegans* WS220 genome version to generate SAM files. SAM files were converted to BAM files also in Galaxy [77]. Binding peaks from ChIP-Seq data (BAM files) were called using SeqSolve software, which uses the MACS algorithm to identify ChIP peaks [39].

### RNA sequencing

Wild type synchronized L1 worms [25] were fed on *rsr-2*, *prp-8* and *gfp* dsRNA-expressing bacteria at 25°C. After 26 hours, the three populations were harvested and frozen with TRIReagent. Total RNA was purified, including small RNAs, using the mirVana miRNA isolation kit (Ambion). Ribosomal RNA was depleted with the RiboMinus Eukaryote Kit (Invitrogen). RNA quality and integrity were evaluated with the Experion Bioanalyzer (Bio-Rad). Libraries for sequencing were made by using the Illumina TruSeq RNA Sample Preparation Kit. These libraries were run through a Genome Analyzer IIx Ultrasequencer (Illumina). Each sample yields more than 10 millions reads. The resulting fastq files were trimmed and mapped to the version WS225 of the *C. elegans* genome by using TopHat to generate BAM files that were analyzed in SeqSolve under the default settings (false discovery rate of 0.05). Reads displaying multiple mapping were filtered out. SeqSolve was used to perform a differential transcript expression analysis between *rsr-2(RNAi)* and *gfp(RNAi)* samples, and between *prp-8(RNAi)* and *gfp(RNAi)* samples. Transcripts covered by more than 5 reads were tested. This analysis uses Cufflinks/Cuffdiff [80] to quantify and identify transcripts with a significant level of expression between different conditions. In this analysis, expression values were normalized in FPKM (fragments per kilobase of exon per million fragments mapped). To analyze intron retention, a file containing intron sequences (excluding those introns with internal genes or ncRNAs) was used as a reference genome.

### Immunoprecipitation

N2 worms were recovered from plates and washed three times with M9 buffer and twice with a buffer containing 50 mM HEPES-KOH pH 7.6, 1 mM EDTA, 140 mM KCL, 0.5% NP40 and 1% glycerol. Worms resuspended in the same buffer (proteases and phosphatases inhibitors added) were added dropwise into liquid nitrogen and worm-pearls were ground to a fine powder in liquid nitrogen using a pestle and mortar. Worm powder was thawed on ice and lysate passed through a 20-gauge syringe prior to a 2-minute centrifugation at 16,000 g and 4°C. Protein was quantified and immunoprecipitated with the Invitrogen immunoprecipitation kit (143-21D) applying minor modifications (see supporting Text S1 for details). For western blot, samples were resuspended in Laemli buffer and boiled for 5 minutes at 95°C. For proteomic assays, complexes were eluted and precipitated overnight with cold acetone. Antibodies used were: unspecific rabbit IgG (Santa Cruz sc-2027), anti-RSR-2 Q5092 (Sdix), phospho Ser-2 RNAPII A300-654A and phospho Ser-5 RNAPII A300-655A (Bethyl laboratories Inc).

### Two-dimensional electrophoresis, silver staining and peptide digestion

Protein pellet samples were resuspended in rehydration buffer (7 M urea, 2 M thiourea, 2% CHAPS, DTT 80 mM, 0.5% IPG pH 3–10 buffer (GE)), brought up to a final volume of 130 µl, and loaded onto Immobiline DryStrip pH 3–10 7 cm (GE). Second-dimension electrophoresis SDS-PAGE was performed with a 7.5% polyacrylamide gel for 75 minutes at constant voltage (100 V; Mini Protean Hoefer system, GE). Gels were stained with a modified short silver nitrate stain (2), scanned with a GS 8000 Calibrated Densitometer (Bio-Rad), and the images obtained were processed and analyzed with Quantity One (Bio-Rad) image analysis software. Spots of interest were selected, manually excised from gels and subjected to in-gel digestion.

### MudPIT

Precipitated proteins were dissolved in 50 µl of resuspension buffer (6 M urea, 200 mM ammonium bicarbonate), reduced with dithiothreitol (0.2 µM, 1 hour, 37°C) and alkylated in the dark with iodoacetamide (0.4 µM, 30 minutes, 25°C). The protein mixture was then diluted six-fold with 200 mM ammonium bicarbonate and digested with 6 µg of trypsin (Promega, cat # V5113) overnight at 37°C. Samples were acidified with formic acid and cleaned up on a home-made Empore C18 column (3M, St. Paul, MN, USA) [81].

### LC-MS/MS

Samples were subjected to liquid chromatography-tandem mass spectrometry and data were acquired with Xcalibur software. Data were processed using Proteome Discoverer beta version 1.3.0.339 (see supporting Text S1 for details).

## Supporting Information

Figure S1 **Genetic regulatory network of the germline and somatic sex determination.** Uppercase and lowercase names represent proteins and mRNAs, respectively. Positive interactions are shown by arrows and negative interactions are represented by bars. (A) Germline sex determination pathway. Proteins and mRNAs that promote oogenesis and spermatogenesis are indicated in red and blue, respectively. (B) Somatic sex determination pathway. Feminizing factors are shown in red and masculinizing factors in blue (Modified from Zarkower (2006) [28] and Ellis and Schedl (2007) [27]).
(TIF)

Figure S2 ***rsr-2*** **is involved in** ***fem-3*** **3′UTR-mediated repression in intestinal cells.** (A) Transgenic line *qIS43*[*lacZ::fem-3(+)* 3′UTR] fed with *gfp(RNAi)* (top) and *rsr-2(RNAi)* (bottom). X-gal staining is visible in intestinal nuclei upon *rsr-2* RNAi. (B) Reporter transgenes used as positive control and for staining specificity are [*lacZ::fem-3(q96gf)* 3′UTR] and [*lacZ::tra-2(+)* 3′UTR] respectively. (+) more than 80% of animals have >20 intestinal nuclei with X-gal staining. (−) fewer than 20% of the animals have >10 intestinal nuclei with X-gal staining.
(TIF)

Figure S3 **Mitosis-to-meiosis switch and meiotic progression in** ***rsr-2(RNAi)*** **animals.** *rrf-1(pk1417); glp-1(oz264)* and *rrf-1(pk1417); gld-3(q730)* mutants fed from L1stage with RNAi specific for *gfp*, *lsm-2* and *rsr-2* were grown and scored as described elsewhere [56]. More than 70 germlines were scored for each condition. Horizontal axis indicates the percentage of animals displaying each phenotype. Additional information can be found in Text S2 file.
(TIF)

Figure S4 **Germline and spermatogenesis genes are enriched in the list of downregulated genes obtained by tiling arrays of** ***rsr-2(RNAi)*** **animals.** ^1^Germline-specific genes. Union of germline-enriched and germline SAGE (tag>0) [31], intersected with SMD (Strictly Maternal Degradation) class genes [83], subtracted any gene also expressed (tag>0) in muscle, gut or neuron SAGE [84], [85]. Compiled by Andreas Rechtsteiner and Susan Strome. ^2^Spermatogenesis genes [31]. ^3^Soma-specific genes (gut, muscle or neuron). Expressed in gut, muscle, or neuron SAGE (tag>8) minus any gene germline-enriched or germline-expressed (germline SAGE tag>0) [31], [84], [85]. ^4^Germline-enriched genes, not including spermatogenesis-related genes [31]. ^5^Germline-expressed genes based on SAGE data [85]. ^6^Intron retention in alternative Splicing events [47]. ^7^Genes in operons [86]. ^8^Extracted from www.wormbase.org (WS220)
(DOCX)

Figure S5 **Specificity of RSR-2 antibodies in western blot and immunostaining.** (A) Detection of RSR-2 protein levels in control (empty vector) and *rsr-2(RNAi)* animals. Protein extraction was accomplished using 2% SDS. Western blot was performed with specific antibodies for RSR-2: Q5091 and Q5092. Actin antibody C4 was used as a loading control. Arrows indicate three unspecific bands that do not disappear upon *rsr-2* RNAi. (B) Detection of RSR-2 protein in wild type N2 and *rsr-2(tm2607)* animals. Protein was extracted using 1% SDS. Western blot was performed with the specific antibody of RSR-2, Q5092. The truncated protein lacks 65 amino acids, resulting in a protein 7 kDa smaller than the wild type. (C) Immunostaining with anti-RSR-2 (Q5092) and anti-GFP (A11120) and counterstained with DAPI. Upper worm is *tm2625* homozygous. Bottom worm is *tm2625* heterozygous. RSR-2 is not expressed in homozygous *tm2625* arrested larvae in contrast to the expression detected in heterozygous *tm2625* larvae.
(TIF)

Figure S6 **RSR-2 interacts with RNAPII.** RNAPII phosphoisoforms detection by western blot in wild type and *rsr-2(RNAi)* worms with the 8WG16 antibody. POL IIo is the abbreviation for the hyperphosphorylated form of the RNAPII whereas POL IIa represents the hypophosphorylated form of the RNAPII. Hyperphosphorylated RNAPII accumulates in *rsr-2(RNAi)* worms. Actin antibody C4 was used as a loading control.
(TIF)

Figure S7 **Anti-RSR-2 immunoprecipitates chromatin of intronless genes.** Venn diagram representing genes showing peaks in both RNAPII (blue) and RSR-2 (yellow) ChIP-Seq. A third intersection highlights the existence of 42 intronless genes (green) in which both proteins present a peak in the ChIP-Seq.
(TIF)

Figure S8 **Proposed model for** ***rsr-2*** **function in the regulation of the sperm-to-oocyte switch.** (A) Sperm is produced when high levels of FEM-3 are achieved. Translational repressive forces by FBF-1,2 and NOS-3 proteins are weak. (B) Oocytes are produced when FEM-3 levels are low. Translational repressive forces by FBF-1,2 and NOS-3 proteins are strong. (C) In *rsr-2(RNAi)* worms, in which most germline genes are downregulated, levels of *fem-3* mRNA are low, but levels of FBFs and NOS-3 proteins are also low. In this case, such global deregulation leads to the complete translation of the available *fem-3* mRNA, reaching the required threshold of FEM-3 to maintain the sperm-to-oocyte switch off.
(TIF)

Figure S9 **Balance between splice forms expressed at L3 after** ***rsr-2*** **and** ***prp-8*** **RNAi.** RNA-Seq data were analyzed with the SeqSolve software to quantify the reads in several transcripts from genes displaying alternative splicing at the L3 stage [47]. Values for each transcript are represented as Fragments Per Kilobase of exon per Million fragments mapped (FPKM).
(TIF)

Figure S10 **ChIP-Seq peak profiles of RNAPII and RSR-2 at five gene loci.** RNAPII peaks are represented in black, RSR-2 peaks are represented in red, and input samples are represented in blue. In all cases RNAPII accumulates at the 5′ gen ends upon *rsr-2(RNAi)*.
(TIF)

Table S1 **Tiling arrays analyses of** ***rsr-2(RNAi) vs. gfp(RNAi)*** **L4 worms.**
(XLSX)

Table S2 **Called peaks and corresponding genes from ChIP-Seq assays with anti-RNAPII and anti-RSR-2, and list of intronless genes.**
(XLSX)

Table S3 **RNA-Seq data. Genes down and upregulated in** ***rsr-2(RNAi)*** **and** ***prp-8(RNAi)*** **.**
(XLSX)

Table S4 **Mass spectrometry analysis of RSR-2-containing complexes.**
(XLSX)

Text S1 **Expanded Material and Methods.**
(DOCX)

Text S2 **Supplementary information for Supporting Figure S3.**
(DOCX)
