# Supplementary figures and images for: RSR-2, the Caenorhabditis elegans Ortholog of Human Spliceosomal Component SRm300/SRRM2, Regulates Development by Influencing the Transcriptional Machinery
Source: PLoS Genet. 2013 Jun 6;9(6):e1003543. doi: 10.1371/journal.pgen.1003543 (PMC3675011; doi:10.1371/journal.pgen.1003543)

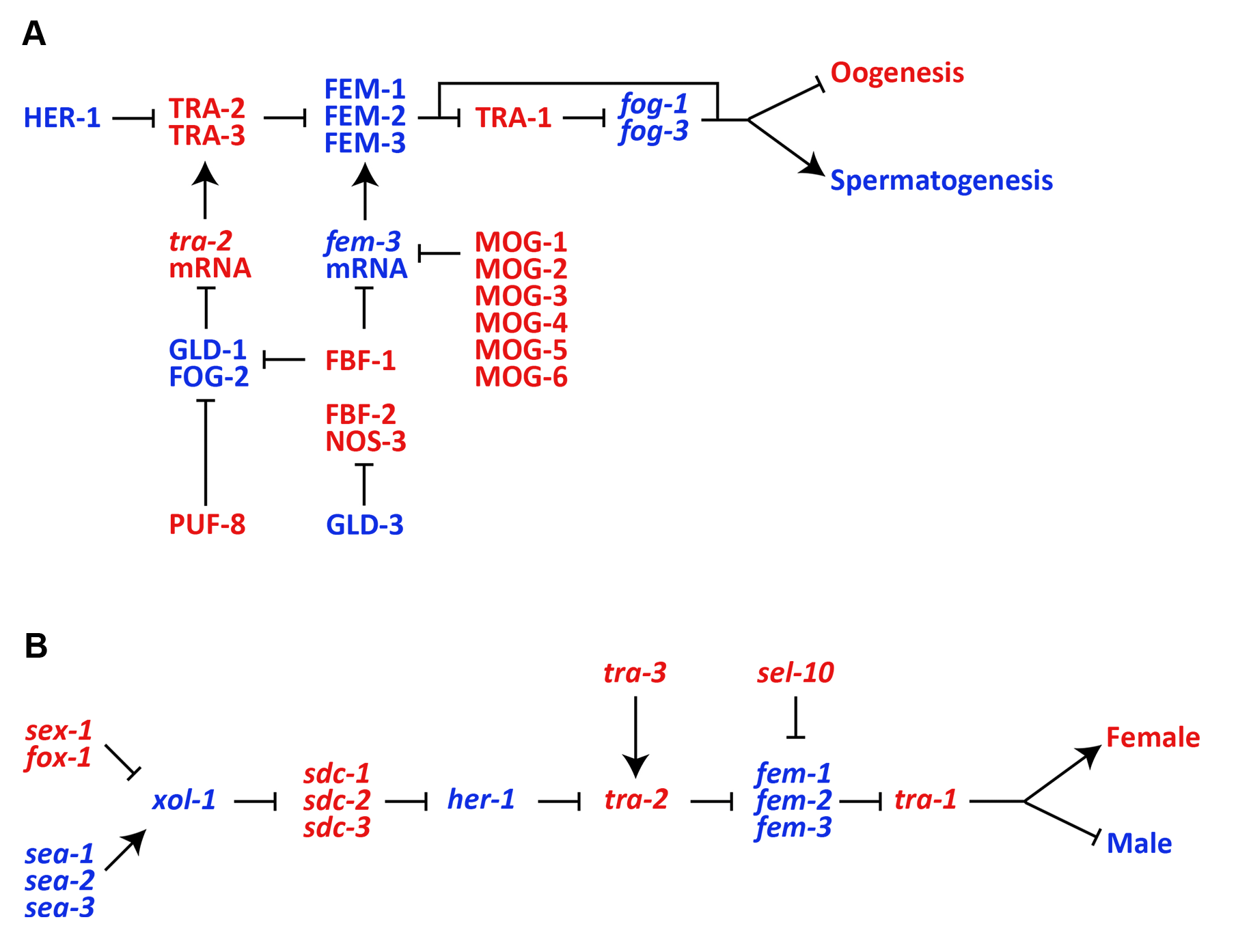

Supplement: Figure S1 — Genetic regulatory network of the germline and somatic sex determination. Uppercase and lowercase names represent proteins and mRNAs, respectively. Positive interactions are shown by arrows and negative interactions are represented by bars. (A) Germline sex determination pathway. Proteins and mRNAs that promote oogenesis and spermatogenesis are indicated in red and blue, respectively. (B) Somatic sex determination pathway. Feminizing factors are shown in red and masculinizing factors in blue (Modified from Zarkower (2006) [28] and Ellis and Schedl (2007) [27]). (TIF) [file pgen.1003543.s001.tif]

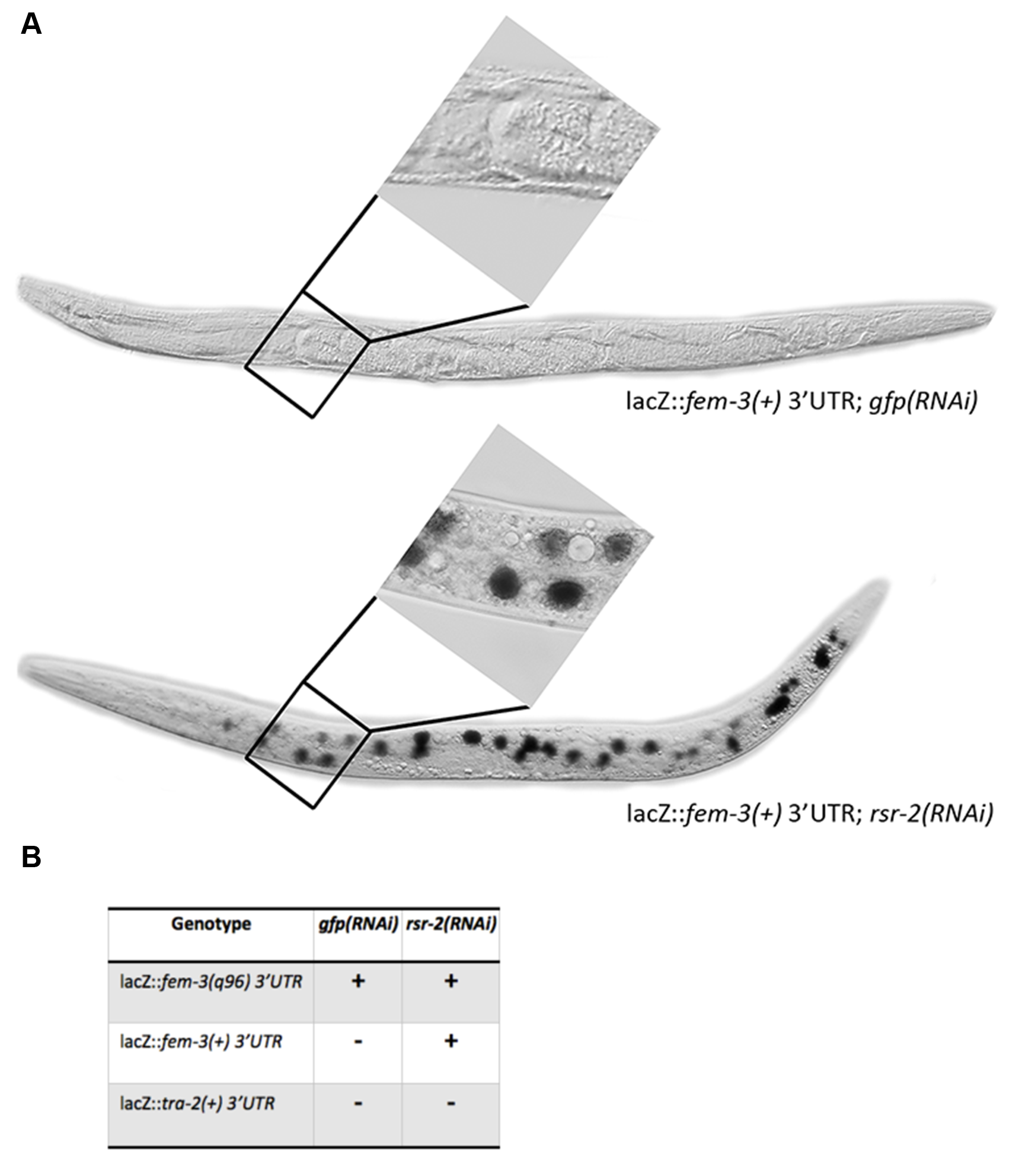

Supplement: Figure S2 — rsr-2 is involved in fem-3 3′UTR-mediated repression in intestinal cells. (A) Transgenic line qIS43[lacZ::fem-3(+) 3′UTR] fed with gfp(RNAi) (top) and rsr-2(RNAi) (bottom). X-gal staining is visible in intestinal nuclei upon rsr-2 RNAi. (B) Reporter transgenes used as positive control and for staining specificity are [lacZ::fem-3(q96gf) 3′UTR] and [lacZ::tra-2(+) 3′UTR] respectively. (+) more than 80% of animals have >20 intestinal nuclei with X-gal staining. (−) fewer than 20% of the animals have >10 intestinal nuclei with X-gal staining. (TIF) [file pgen.1003543.s002.tif]

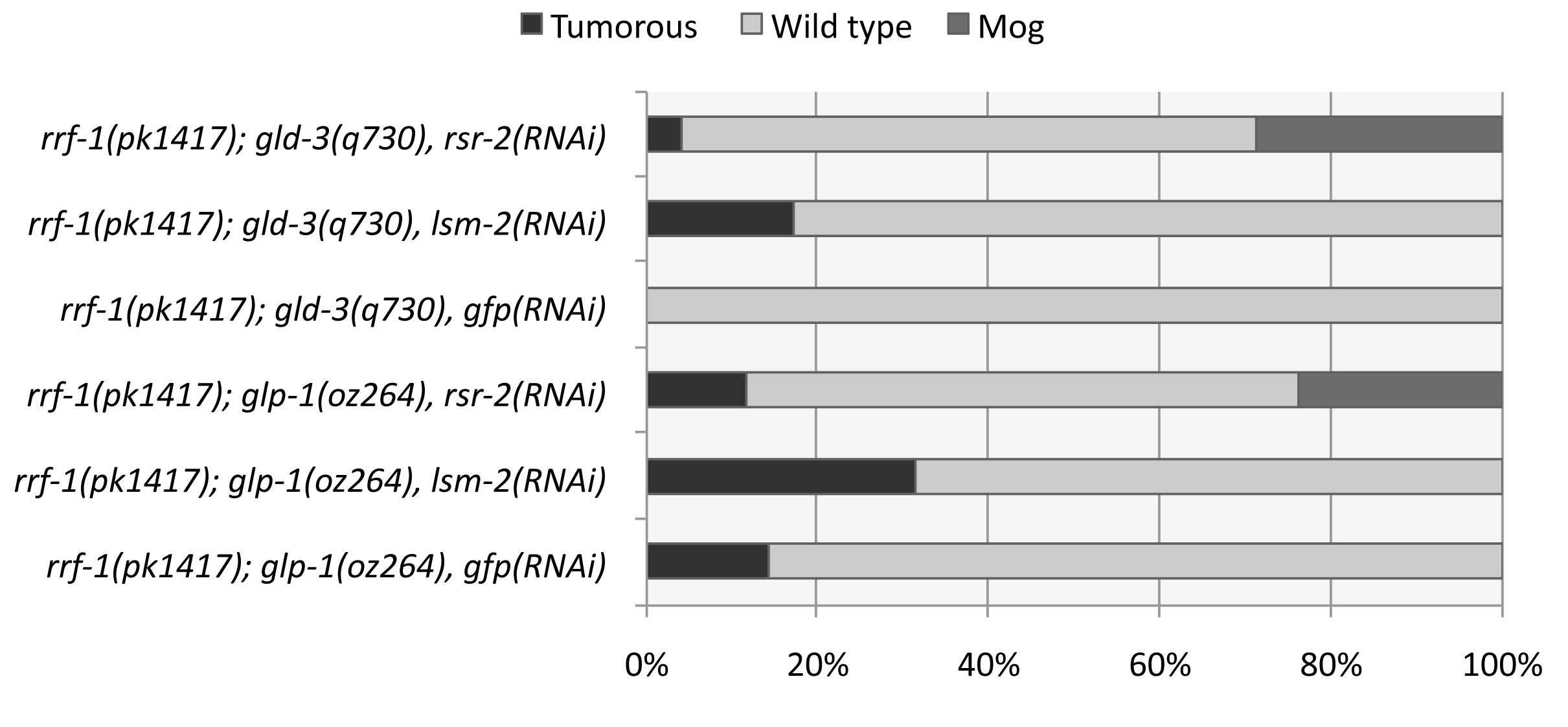

Supplement: Figure S3 — Mitosis-to-meiosis switch and meiotic progression in rsr-2(RNAi) animals. rrf-1(pk1417); glp-1(oz264) and rrf-1(pk1417); gld-3(q730) mutants fed from L1stage with RNAi specific for gfp, lsm-2 and rsr-2 were grown and scored as described elsewhere [56]. More than 70 germlines were scored for each condition. Horizontal axis indicates the percentage of animals displaying each phenotype. Additional information can be found in Text S2 file. (TIF) [file pgen.1003543.s003.tif]

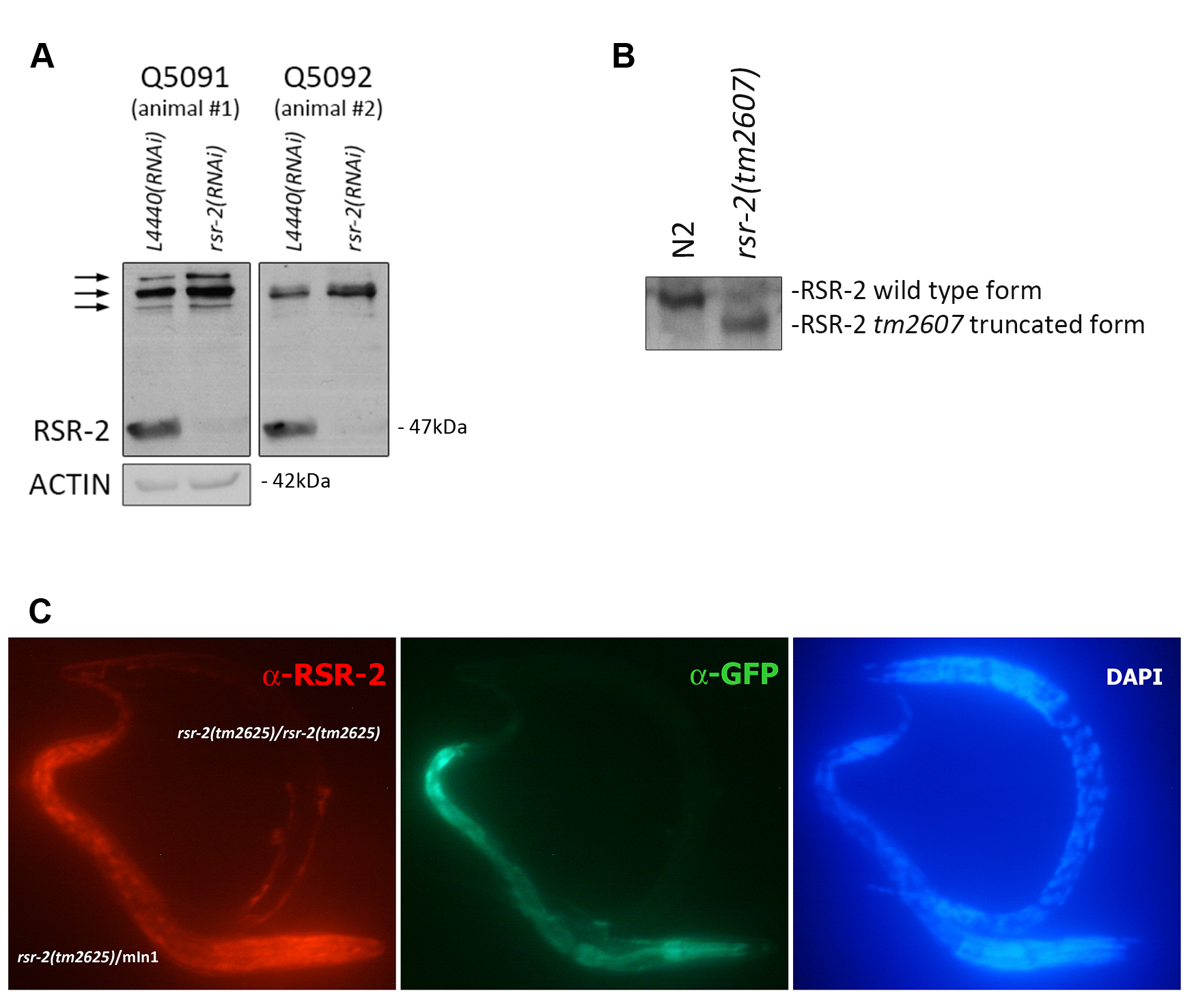

Supplement: Figure S5 — Specificity of RSR-2 antibodies in western blot and immunostaining. (A) Detection of RSR-2 protein levels in control (empty vector) and rsr-2(RNAi) animals. Protein extraction was accomplished using 2% SDS. Western blot was performed with specific antibodies for RSR-2: Q5091 and Q5092. Actin antibody C4 was used as a loading control. Arrows indicate three unspecific bands that do not disappear upon rsr-2 RNAi. (B) Detection of RSR-2 protein in wild type N2 and rsr-2(tm2607) animals. Protein was extracted using 1% SDS. Western blot was performed with the specific antibody of RSR-2, Q5092. The truncated protein lacks 65 amino acids, resulting in a protein 7 kDa smaller than the wild type. (C) Immunostaining with anti-RSR-2 (Q5092) and anti-GFP (A11120) and counterstained with DAPI. Upper worm is tm2625 homozygous. Bottom worm is tm2625 heterozygous. RSR-2 is not expressed in homozygous tm2625 arrested larvae in contrast to the expression detected in heterozygous tm2625 larvae. (TIF) [file pgen.1003543.s005.tif]

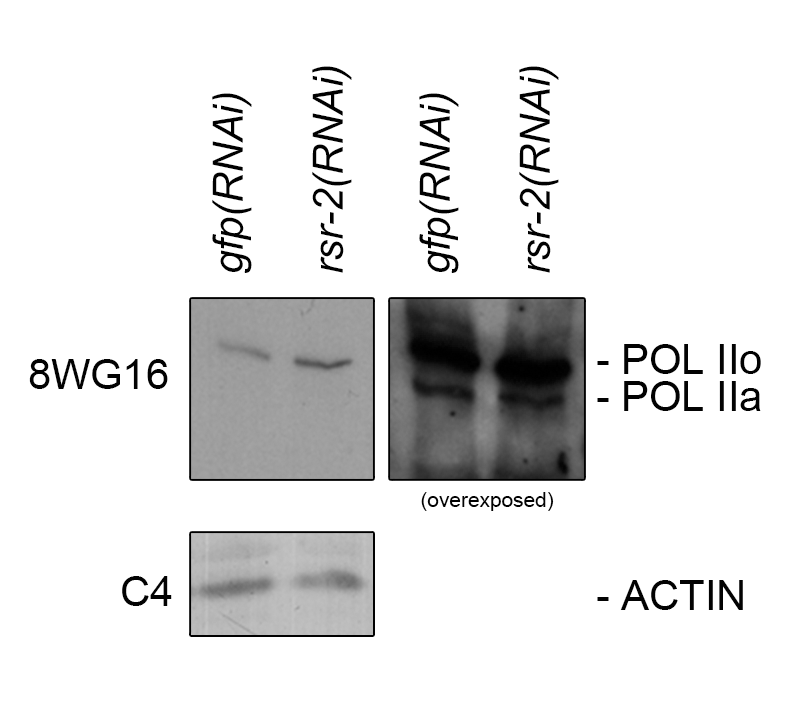

Supplement: Figure S6 — RSR-2 interacts with RNAPII. RNAPII phosphoisoforms detection by western blot in wild type and rsr-2(RNAi) worms with the 8WG16 antibody. POL IIo is the abbreviation for the hyperphosphorylated form of the RNAPII whereas POL IIa represents the hypophosphorylated form of the RNAPII. Hyperphosphorylated RNAPII accumulates in rsr-2(RNAi) worms. Actin antibody C4 was used as a loading control. (TIF) [file pgen.1003543.s006.tif]

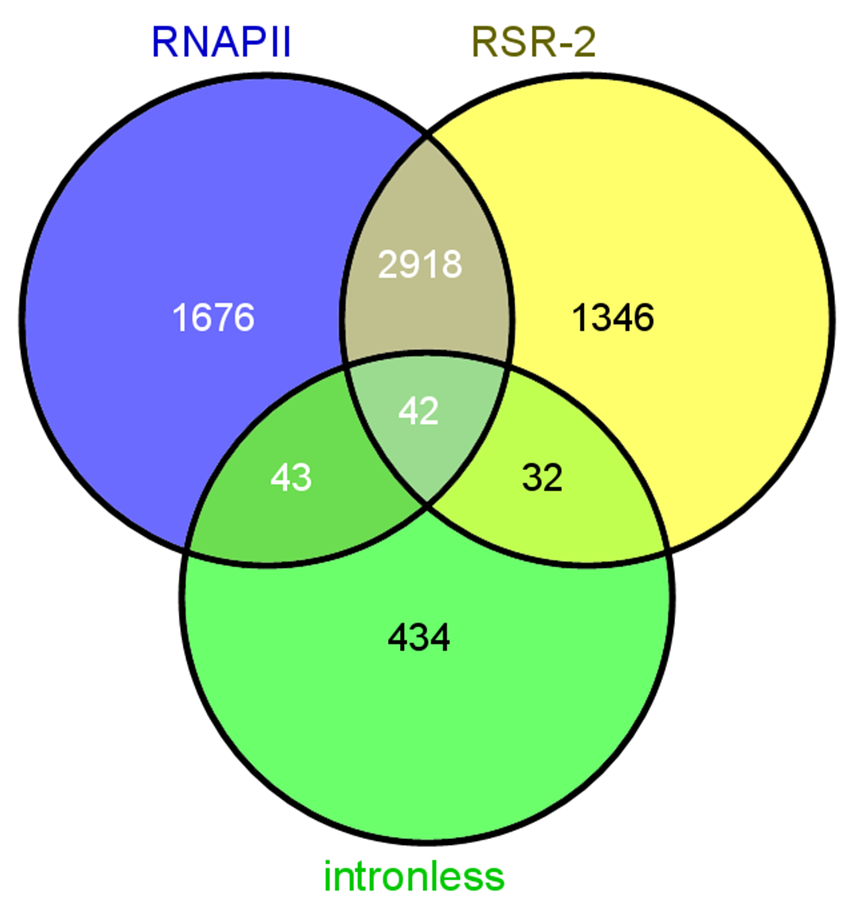

Supplement: Figure S7 — Anti-RSR-2 immunoprecipitates chromatin of intronless genes. Venn diagram representing genes showing peaks in both RNAPII (blue) and RSR-2 (yellow) ChIP-Seq. A third intersection highlights the existence of 42 intronless genes (green) in which both proteins present a peak in the ChIP-Seq. (TIF) [file pgen.1003543.s007.tif]

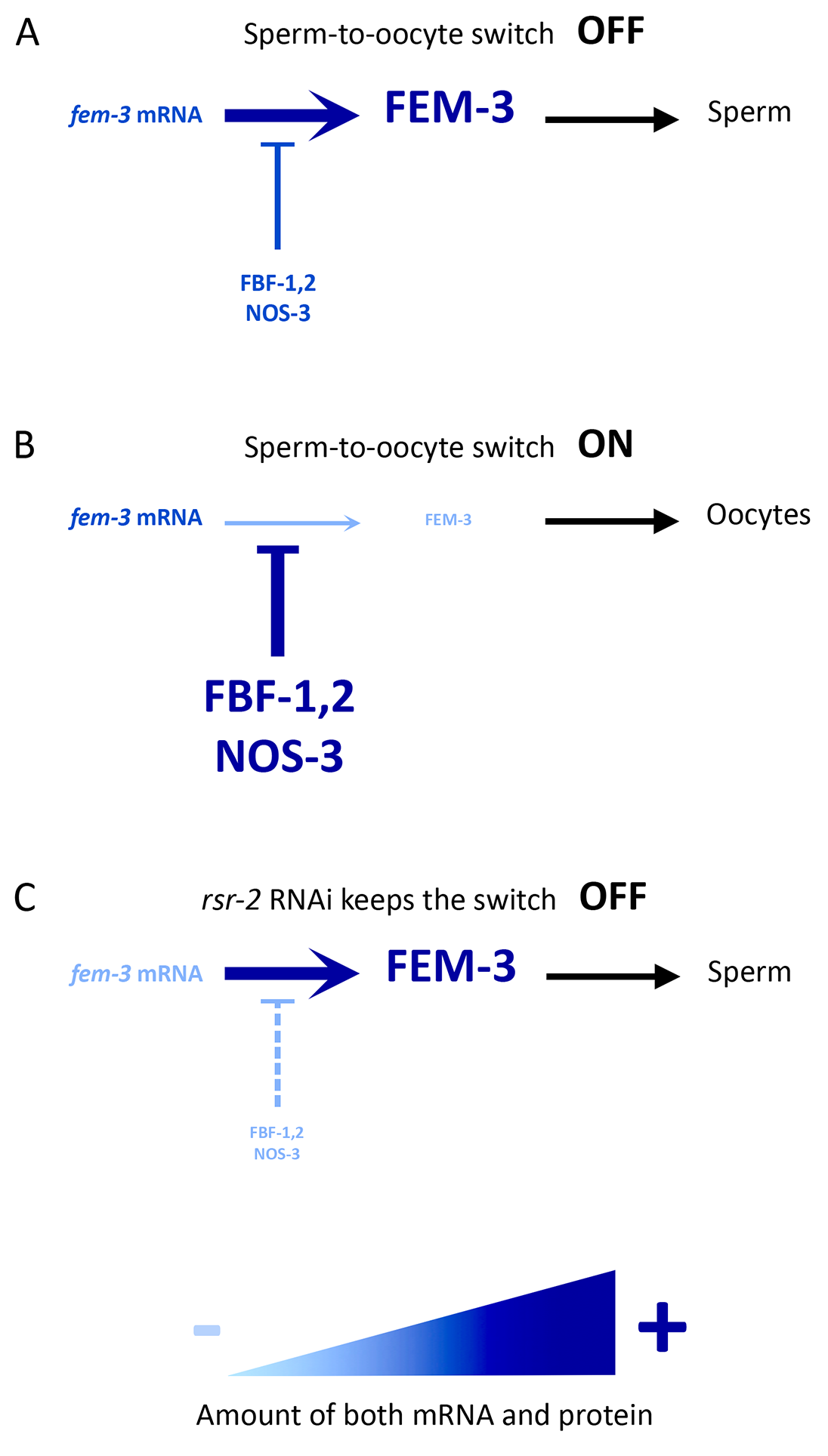

Supplement: Figure S8 — Proposed model for rsr-2 function in the regulation of the sperm-to-oocyte switch. (A) Sperm is produced when high levels of FEM-3 are achieved. Translational repressive forces by FBF-1,2 and NOS-3 proteins are weak. (B) Oocytes are produced when FEM-3 levels are low. Translational repressive forces by FBF-1,2 and NOS-3 proteins are strong. (C) In rsr-2(RNAi) worms, in which most germline genes are downregulated, levels of fem-3 mRNA are low, but levels of FBFs and NOS-3 proteins are also low. In this case, such global deregulation leads to the complete translation of the available fem-3 mRNA, reaching the required threshold of FEM-3 to maintain the sperm-to-oocyte switch off. (TIF) [file pgen.1003543.s008.tif]

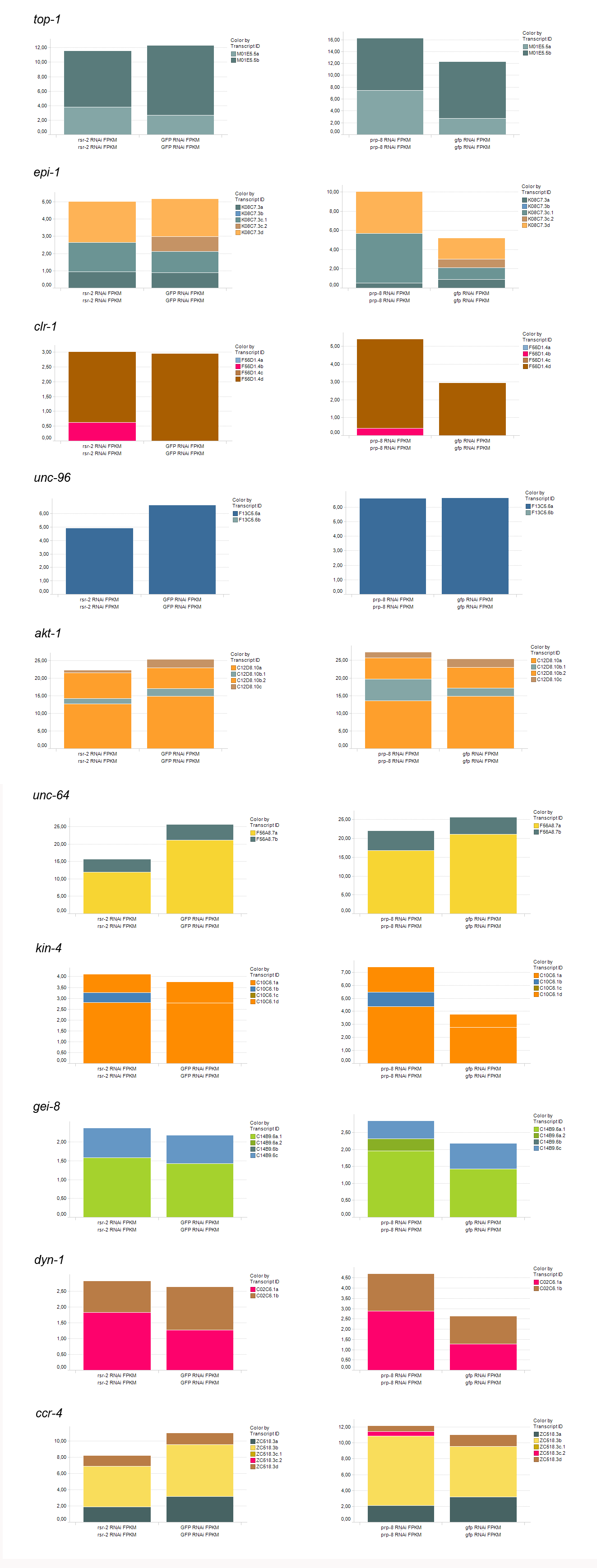

Supplement: Figure S9 — Balance between splice forms expressed at L3 after rsr-2 and prp-8 RNAi. RNA-Seq data were analyzed with the SeqSolve software to quantify the reads in several transcripts from genes displaying alternative splicing at the L3 stage [47]. Values for each transcript are represented as Fragments Per Kilobase of exon per Million fragments mapped (FPKM). (TIF) [file pgen.1003543.s009.tif]

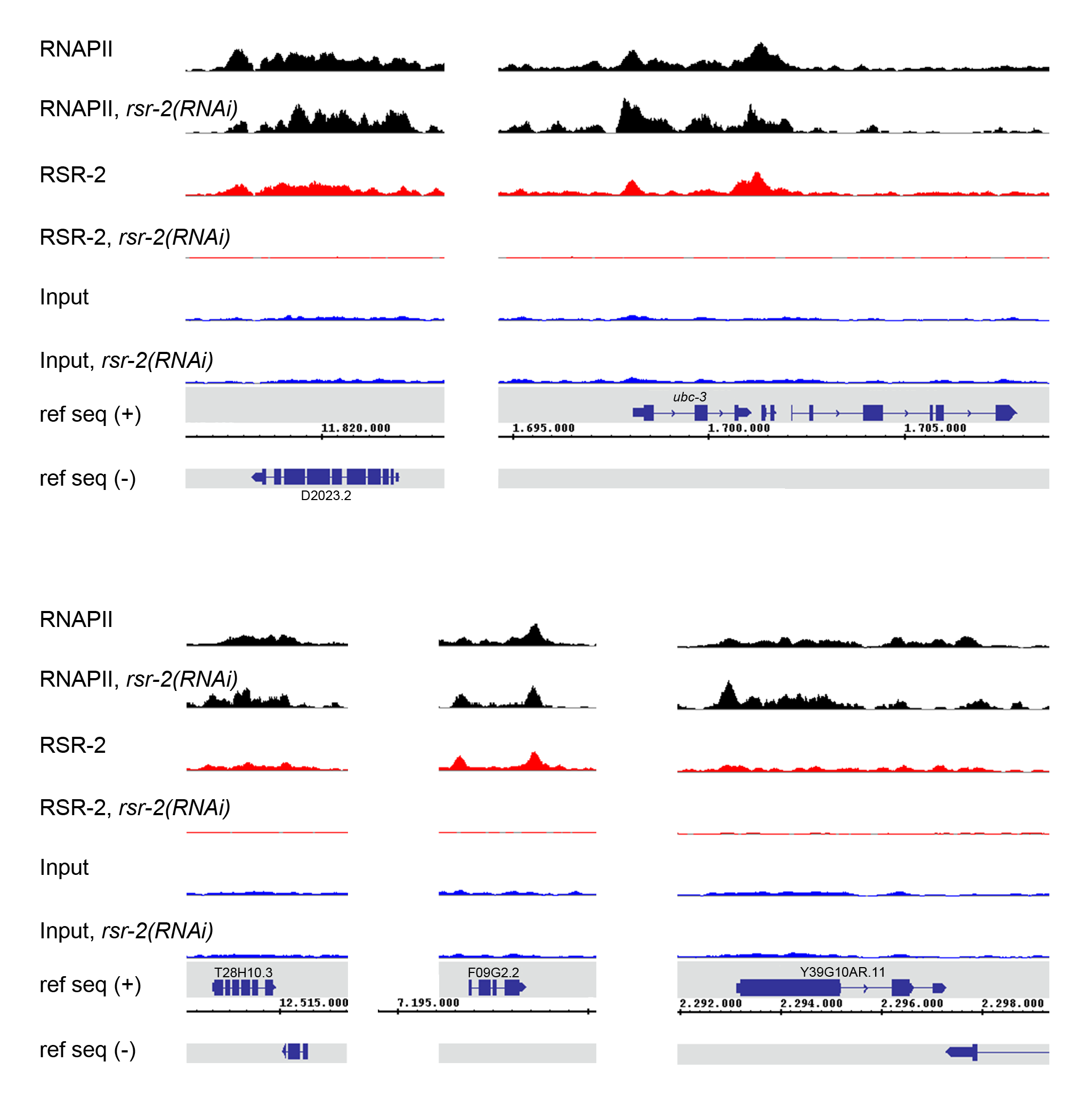

Supplement: Figure S10 — ChIP-Seq peak profiles of RNAPII and RSR-2 at five gene loci. RNAPII peaks are represented in black, RSR-2 peaks are represented in red, and input samples are represented in blue. In all cases RNAPII accumulates at the 5′ gen ends upon rsr-2(RNAi). (TIF) [file pgen.1003543.s010.tif]
