# Supplementary material for: RSR-2, the Caenorhabditis elegans Ortholog of Human Spliceosomal Component SRm300/SRRM2, Regulates Development by Influencing the Transcriptional Machinery
Source: PLoS Genet. 2013 Jun 6;9(6):e1003543. doi: 10.1371/journal.pgen.1003543 (PMC3675011; doi:10.1371/journal.pgen.1003543)
Supplement: Figure S4 — Germline and spermatogenesis genes are enriched in the list of downregulated genes obtained by tiling arrays of rsr-2(RNAi) animals. 1Germline-specific genes. Union of germline-enriched and germline SAGE (tag>0) [31], intersected with SMD (Strictly Maternal Degradation) class genes [83], subtracted any gene also expressed (tag>0) in muscle, gut or neuron SAGE [84], [85]. Compiled by Andreas Rechtsteiner and Susan Strome. 2Spermatogenesis genes [31]. 3Soma-specific genes (gut, muscle or neuron). Expressed in gut, muscle, or neuron SAGE (tag>8) minus any gene germline-enriched or germline-expressed (germline SAGE tag>0) [31], [84], [85]. 4Germline-enriched genes, not including spermatogenesis-related genes [31]. 5Germline-expressed genes based on SAGE data [85]. 6Intron retention in alternative Splicing events [47]. 7Genes in operons [86]. 8Extracted from www.wormbase.org (WS220) (DOCX) [file pgen.1003543.s004.docx]

| **Gene clasess** | **Upregulated > 1,2**  **1609** | **Downregulated < 0,8**  **2308** |
| --- | --- | --- |
| **169 germline-specific genes^1^** | 0 | **28** |
| **844 spermatogenesis genes^2^** | 1 | **298** |
| 1177 soma specific genes^3^ | 36 | 26 |
| **2215 germline-enriched genes^4^** | 34 | **133** |
| **4678 germline-expressed^5^** | 38 | **138** |
| 545 intron-retention AS^6^ | 10 | 16 |
| 3339 genes in operons^7^ | 35 | 41 |
| 551 intronless genes^8^ | 48 | 64 |
