# Supplementary material for: RSR-2, the Caenorhabditis elegans Ortholog of Human Spliceosomal Component SRm300/SRRM2, Regulates Development by Influencing the Transcriptional Machinery
Source: PLoS Genet. 2013 Jun 6;9(6):e1003543. doi: 10.1371/journal.pgen.1003543 (PMC3675011; doi:10.1371/journal.pgen.1003543)
Supplement: Text S1 — Expanded Material and Methods. (DOCX) [file pgen.1003543.s015.docx]

**TEXT S1: Expanded Material and Methods**

**Semiquantitative RT-PCR.**

Worm pellets were washed twice with M9 buffer. Seven volumes of TRIReagent (TR-118, MRC) were added to the packed pellets and were frozen at -80°C. Next, 5 cycles of freezing/thawing were performed and samples were vortexed vigorously. The suspension was allowed to stand at RT for 5 minutes. 0.2 ml of chloroform per ml of Trizol was added followed by an incubation of 15 minutes at RT. The aqueous phase was taken and RNA was precipitated with isopropanol and washed ethanol. RNA was resuspended in DEPC-treated water and its quality and quantity determined by running an agarose gel and taking spectrophotometric readings. cDNA was synthesized from 1 μg of purified RNA with oligo(dT) primers using the RevertAid H Minus First Strand cDNA synthesis kit (Fermentas. Cat.No. K1632) following the manufacturer’s instructions. Sequences of primers used in the RT-PCR assay can be found at the end of this section.

***In situ* hybridization of mRNA**

DNA probes were labeled with digoxigenin-dUTP by repeated primer extension (DIG DNA Labeling Mix. Roche Cat. No. 11 277 065 910). Gonads from adult hermaphrodites dissected in a multi-well Pyrex plate (Electron Microscopy Sciences, Cat. 71561-01) were permeabilized (50 μg/ml proteinase K) for 30 minutes at RT and fixed (3% paraformaldehyde / 0.25% glutaraldehyde / 0.1M K_2_HPO_4_) for 2 hours at RT. Both sense and antisense probes were diluted 5 times in hybridization buffer and hybridized for 36 hours at 48°C. Probe detection was carried out by incubating the samples with a 400-fold-diluted alkaline-phosphatase conjugated anti-DIG antibody overnight at 4°C. BCIP/NDT (Sigma Cat. No. B5655) was used to set up the colorimetric reaction, dissolved in staining solution. Gonads were counterstained with DAPI (2 μg/ml), embedded in anti-fade solution (Invitrogen Cat. No. P36930) and mounted on a microscope slide.

**Immunofluorescence**

A) Of dissected gonads

Dissected gonads were fixed with 4% paraformaldehyde (Electron Microscopy Sciences Cat. No. 15710) for 20 minutes and subsequently washed 3 times with PBS and 0.1% Tween-20 (PBSt) (10 minutes each wash). Pre-incubation with PBSt and BSA (1 mg/ml) (Sigma Cat. No. A9418) was carried out for 1 hour at RT. After it, the epitope of interest was detected by incubating with the corresponding primary antibody overnight at 4°C in a sealed humidity chamber. The following day, gonads were left 1 hour at RT and washed three times with PBSt plus BSA (1 mg/ml). Secondary Antibodies were incubated for 2 hours at RT. Finally, the antibody was washed out and gonads were counterstained with DAPI (2 μg/ml), embedded in anti-fade solution (Invitrogen Cat. No. P36930) and mounted in a glass microscope slide.

B) Of larvae (Freeze–Cracking protocol)

For immunostaining of larvae, freeze-cracking protocol was followed as described by Duerr et al, 2006 [1]. This protocol provides a simple way to remove portions of the worm’s cuticle allowing its penetration by antibodies. Slides were incubated 10 minutes in Poly-L-lysine 0.1% (dilution 1:10) (SIGMA, P8920) and baked at 60°C for 15 minutes. In parallel, larvae were washed using PBS. Once the polylysine coated slides were dried and chilled to RT, around 50 worms were placed on each slide, allowed to settle and stick on the surface for a few minutes. Excess of liquid was removed with a pipette and a coverslip was set so that the edge of the coverslip extended over the edge of the slide. Pressure on the coverslip was put with the fingertips and the slides were placed on a prefrozen aluminum block on dry ice. Slides were left on the cold block until they were frozen. Coverslips were popped off and the slides immediately soaked in prechilled (-20°C) 100% methanol for 15 minutes, followed by 10 minutes in prechilled (-20°C) 100% acetone and three washing steps (10 minutes each) with PBSt plus 10 mg/ml BSA. After a 30-minute pre-incubation with PBSt plus 10mg/ml BSA, 50 μl of the primary antibody of interest was applied, the slide covered with a parafilm to avoid evaporation and provide homogenous distribution of the antibody, and incubated at 4°C overnight in a humidity chamber. Next day, three PBSt washing steps of ten minutes each were carried out and 50 μl of the secondary antibody were spread over the fixed worms. Samples were left 2 hours at RT and after three more washing steps with PBSt (10 minutes each), samples were embedded with anti-fade solution and counterstained with DAPI (2 μg/ml).

**Gateway cloning**

The pENTRY vector containing *rsr-2* genomic and 3’UTR fragment was digested with PvuII before the LR reaction, which significantly improved the efficiency of the LR reaction. Transformation of Library Efficiency DH5α competent cells (Invitrogen Cat. No. 18263-012) was performed with 100μg/ml ampicillin selection in solid cultures. All the obtained Entry and Expression clones were verified by sequencing.

**Protein analysis**

Wild type animals were harvested after 36 hours post-L1 arrest at 25°C and washed in M9 buffer. After 30-minute incubation in M9 buffer with agitation to get rid of the remaining bacteria, worms were packed and frozen. Worm lysis was performed by adding 2 pellet volumes of lysis buffer directly onto frozen worms. Protein from worms was extracted by 3 cycles of freezing in liquid nitrogen and boiling for 15 min. Next, a 10-minute centrifugation at 4ºC was performed and total protein in the lysate was quantified using the Bio-Rad DC protein assay (Bio-Rad Cat. No. 500-0112).

Then, protein was loaded in sodium dodecyl sulfate polyacrylamide gel (SDS-PAGE) at different percentages and gels were run in TGS buffer. Proteins were transferred to a nitrocellulose membrane (Protran, Cat. No. BA85, 0.45 μm) for two hours at 200 mA using transfer buffer. Membranes were blocked with 5 % non-fat milk in TBS-t for 1 hour. Primary antibody was added to fresh blocking solution or prepared in 3 % bovine serum albumin TBSt-3% BSA and incubated O/N at 4ºC. After three ten-minute washing steps with TBSt-milk, secondary antibody peroxidase-combined (HRP) was incubated (in the same solution) for 1 hour at RT. Two additional ten-minute washes were performed with TBSt prior to develop the membranes using a substrate for HRP (luminol). Membranes were incubated with luminol plus enhancer for 1 minute and exposed to autoradiographic films (CL-xposure films, Cultek S.A.).

Antibodies and dilutions used for protein analyses of this study were:

| Antibody | Epitope | Host | Nature | Dilution | Source |
| --- | --- | --- | --- | --- | --- |
| Q5092 | RSR-2 | Rabbit | Polyclonal | 1:500 | Sdix |
| C4 (69100) | Actin | Mouse | Monoclonal | 1:500 | MP biomedicals |
| 10799 | Phospho Histone 3 | Mouse | Monoclonal | 1:2000 | mAbcam |
| 8W16G (MMS-126R) | CTD RNAPII | Mouse | Monoclonal | 1:500 | Covance |
| N-20 (sc-899) | N-ter RNAPII | Rabbit | Polyclonal | 1:1000 | Santa Cruz Biotech |
| P0260 | Anti-mouse | Rabbit | Polyclonal | 1:2000 | Dako |
| P0448 | Anti-rabbit | Goat | Polyclonal | 1:2000 | Dako |
| A300-654A | Phospho Ser-2 RNAPII | Rabbit | Polyclonal | 1:5000 | Bethyl Laboratories |
| A330-655A | Phospho Ser-5 RNAPII | Rabbit | Polyclonal | 1:5000 | Bethyl Laboratories |

**ChIP-Seq**

ChIP assays were carried out as described previously by Zhong et al. with minor modifications [2]. Worms were collected at L4 stage and 0.5 ml of packed larvae was resuspended in 3 ml of FA buffer plus protease inhibitors and crosslinked with 2% formaldehyde for 30 minutes at room temperature. Quenching of formaldehyde was carried out by addition of 1M Tris (pH 7.5). Next, samples were sonicated on ice using a Branson sonifier microtip (100% amplitude, 10 seconds on, 10 seconds off). Cell extracts containing DNA fragments with an expected range between 200 and 800 bp were immunoprecipitated using anti-RSR-2 (Q5092) and RNAPII antibodies (8WG16). 2.2 mg of protein were treated as described in Zhong et al [2]. Input samples were set apart and 10 μg of each antibody were added to each sample. ChIP was performed O/N at 4°C with rotation.

Then, 25 μl of protein G conjugated to sepharose beads (Amersham Biosciences) were added to each ChIP sample and washed 4 times with 1 ml FA buffer. After the washes, beads were suspended in 1 bead volume of FA buffer, and 40 μl of the bead slurry was added to each ChIP sample and rotated at 4°C for 2 hours. Next, beads were washed as follows: 2 washes with FA buffer for 5 minutes, 1 wash with FA-1M NaCl for 5 minutes, 1 wash with FA-500mM NaCl for 10 minutes, 1 wash with TEL buffer for 10 minutes, 2 washes with TE buffer for 5 minutes.

To elute the immunocomplexes, 150 μl of elution buffer were added to the samples and the tubes incubated at 65°C for 15 minutes, with brief vortexing every 5 minutes. The beads were spun down and the supernatant transferred to a new tube. The elution was repeated and supernatants combined. At this point, input samples were thawed and treated with the ChIP samples. To each sample, 2 μl 10 mg/ml RNAse A were added and incubated at room temperature for 1–2 hours. Then, 250 μl of elution buffer with 1 μl of 20 mg/ml proteinase K were added to each sample and incubated for 1–2 hours at 55°C. Samples were transferred to 65°C for 12–20 hours to reverse crosslinks. The DNA was purified with the QIAquick PCR purification kit (Qiagen), and eluted with 50 μl H2O. The enriched DNA fragments and input DNA were used to prepare libraries for sequencing by the Illumina GA platform. In order to run four samples in one flow cell, sequencing libraries were barcoded and multiplexed.

Calling binding peaks from ChIP-Seq data was performed using SeqSolve software. First, ChIP-Seq fastq files obtained from the ModEncode consortium were processed in Galaxy and mapped against the *Caenorahabditis elegans* WS220 genome version to generate SAM Files. SAM files were converted to BAM files also in Galaxy [3]. BAM files were analyzed with the SeqSolve software using default settings. In these default settings, the output from the peak caller was filtered by using a False Discovery Rate (FDR) larger than 0.1. Thus, peaks having a FDR larger than 0.1 (10%) were filtered out. Peak calling p-value cutoff was 10e-5. SeqSolve Peak Calling analysis uses the MACS algorithm [4] to identify those regions of the genome having higher read counts in the ChIP samples than in the input sample. At these conditions, 6889, 5451 and 412 peaks were called for anti-RNAP II, anti-RSR-2 and input respectively. The numbers of mapped reads for each ChIP used in this study are showed in the table below.

**Immunoprecipitation**

Asynchronous N2 worms from 4 150 mm NGM plates with 10x OP50 were washed off with M9 and harvested for 2 minutes at 400 g. Pelleted worms were washed three times with M9 buffer and twice with a buffer containing 50 mM HEPES-KOH pH7.6, 1mM EDTA, 140 mM KCL, 0.5% NP40, 1% glycerol. Worms were then resuspended in the same buffer with proteases and phosphatases inhibitors and the worm suspension dripped dropwise in liquid nitrogen to form pearls that were stored at -80^o^C. Worm-pearls were grinded to a fine powder in liquid nitrogen using a mortar and pestle previously cooled with liquid nitrogen. Worm powder was transferred into an eppendorf and thawed on ice. Lysate was then passed through a 20-gauge syringe 10 times and centrifuged for 2 minutes at 16.000 g and 4^o^C.

Supernatant was transferred to a new tube and protein amount quantified. Immunoprecipitation was carried out with the Invitrogen immunoprecipitation kit (143-21D). Briefly, protein extract was precleared with Dynabeads (Invitrogen) for 1h at 4^o^C in agitation. 5 μg of each antibody (unspecific Rabbit IgG Santa Cruz sc-2027, anti-RSR-2 Q5092 Sdix, Phospho RNA Polymerase II (S2) A300-654A Bethyl laboratories Inc. and Phospho RNA Polymerase II (S5) A300-655A Bethyl laboratories Inc.) was combined to Dynabeads according to manufacturer’s instructions and incubated in agitation for 1h at 4^o^C. Precleared extract was then split into the vial with the different antibodies-combined beads (2 mg per IP), volume adjusted to 1 ml with IP buffer (50 mM HEPES-KOH pH7.6, 1 mM EDTA, 250 mM KCL, 0.05% NP40, protease and phosphatase inhibitors) and incubated in agitation for 2 hours at 4^o^C.

Three washes with 1 ml IP buffer and two washes with 200 μl washing buffer (Invitrogen) were performed prior to elution of specific bound complexes. For western blot, samples were resuspended in Laemli buffer and boiled for 5 minutes at 95^o^C. For proteomic assays complexes were eluted with the elution buffer provided with the kit. Elutes were precipitated overnight with 6 volumes of cold acetone and centrifuged for 5 minutes at maximum speed prior to discarding the supernatant and drying the pellet for further processing.

**Two dimensional electrophoresis, silver staining and peptide digestion**

Protein pellet samples were resuspended in rehydration buffer composed of 7 M urea, 2 M thiourea, 2% CHAPS, DTT 80 mM, 0.5% IPG pH 3-10 buffer (GE), brought up to a final volume of 130 µL, and loaded onto Immobiline^TM^ DryStrip pH 3-10 7cm (GE). Immobilized pH gradient strips were subjected to passive rehydration overnight (16h) at 20^o^, and then run on a IPGphor (GE) device following these voltage steps: first step-hold 30´ 300 V, second step-gradient 30’ 1000 V, third step-gradient 1h 30’ 5000V, and a final fourth step-hold 40’ 5000 V reaching a total accumulated 6.5 KVh. The IPG strips were equilibrated twice for 15 min with gentle shaking in 3 mL in a buffer containing 50mM Tris-HCl buffer (pH 8.8), 6 M urea, 30% w/v glycerol, 2% SDS and traces of bromophenol blue. DTT (65 mM) was added to the first and iodoacetamide (135 mM) to the second equilibration step. After equilibration, strips were transferred to the second dimension electrophoresis SDS-PAGE which was performed with a 7.5 % polyacrylamide gel for 1h 15´ and constant voltage (100 V; Mini Protean Hoefer system, GE). To assign molecular weight to the spots, a protein ladder was loaded (BenchMark™ Protein Ladder, Invitrogen).

After 2-DE, the gels were stained with a modified short silver nitrate staining [5], scanned with a GS 8000 Calibrated Densitometer (Bio-Rad) and the obtained images processed and analyzed with software image analysis Quantity One (Bio-Rad).

Spots of interest were selected and manually excised from gels and subjected to in-gel digestion. Briefly, excised spots were cleaned with ammonium bicarbonate (25 mM) and acetonitrile (ACN). Proteins were reduced with DTT (10 mM; 30’, 56^o^C) and alkylated with iodoacetamide (55 mM; 21^o^C, 30‘, in the dark) and, finally, were digested with porcine trypsin in 50 mM ABC pH 8 (Sequence grade modified Trypsin, Promega; 80ng trypsin/sample; 37^o^C, 16h). The resulting peptide mixture were extracted from gel matrix with 10% formic acid (FA) and ACN, and dried-down in a vacuum concentrator.

**LC-MS/MS Analysis of excised spots**

The dried-down peptide mixture from tryptic digestion was resuspended 1% FA and analysed with the nano-UPLC nanoAcquity system (Waters) coupled to a mass spectrometer LTQ-OrbitrapVelos (Thermo Scientific). Injected peptides were trapped on a Symmetry C18^TM^ trap column (5µm 180µm x 20mm; Waters), and were separated by reverse phase chromatography using a C18 reverse phase capillary column (75 μm Øi, 10 cm, nano Acquity, 1.7μm BEH column; Waters). A double-step gradient was run from 0 to 40 % B (A: 0.1% FA; B: 99% ACN, 0.1%FA) over 20 minutes, followed by final gradient from 40% to 60% B over 5 min to elute peptides, with a 250 nL/min column flow rate.

Eluted peptides were subjected to electrospray ionization in an emitter needle (PicoTip^TM^, New Objective) with an applied voltage of 2000V to capillary/needle and 60V to cone, and analyzed in the mass spectrometer. LTQ-OrbitrapVelos instrument performed a full scan MS ranging from 350-5000 m/z with a 60.000 FWHM resolution at 400 m/z. Simultaneously, up to the 10 most abundant peptides from a survey MS scan (minimum intensity of 500 counts) were selected and then fragmented using CID *(collision induced dissociation*) in a ionic tramp (C-TRAP)/collision cell (helium collision gas with 38% normalized energy). Generated raw data were collected with Xcalibur (Thermo, v.2.1.0.1140) and analyzed with Proteome Discoverer (Thermo, v.1.3.0.339) software. Peptide sequence database search was performed against UniProtKB/Swiss-Prot (03/10/2012) by Sequest search engine, included in Proteome Discover, against both target and a decoy spectra database to obtain a false discovery rate (FDR), and thus estimate the number of incorrect peptide-spectrum matches (PSMs) that exceed a given threshold. To improve the sensitivity of previous data base search algorithms, *Percolator* (*semi-supervised learning machine*) was used at constant 0.01% FDR. *Percolator* assigns a *q-value* to each spectrum, which is defined as the minimal FDR at which the identification is deemed correct. These q values are estimated using the distribution of scores from decoy database search, and the q-value threshold was set to <0.01 to validate any peptide. Search parameters included: trypsin as enzyme with 1 missed cleavages; oxidation of methionine and pyro-Glu (N-term Glutamine) as a dynamic modification while alkylation at cysteine was set as fixed modification; precursor and fragment mass tolerance were set to 10 ppm and 0.6 Da, respectively. Only the proteins identified with ≥2 peptides with a defined high confidence were selected.

**MudPIT**

Acetone-cold precipitated proteins were dissolved in 50 µl of resuspension buffer (6 M urea, 200 mM ammonium bicarbonate). Samples were reduced with dithiothreitol (0.2 µM, 1 h, 37°C) and alkylated in the dark with iodoacetamide (0.4 µM, 30 min, 25 ºC). The protein mixture was then diluted 6 fold with 200 mM ammonium bicarbonate and digested with 6 µg of trypsin (Promega, cat # V5113) overnight at 37 ºC. Samples were acidified with formic acid and cleaned up on a homemade Empore C18 column (3M, St. Paul, MN, USA) [6].

Samples were analyzed using a LTQ-Orbitrap XL mass spectrometer (Thermo Fisher Scientific, San Jose, CA, USA) coupled to a nano-HPLC (Agilent Technologies 1200 Series, CA, USA). Peptides were loaded onto a C18 Zorbax pre-column (Agilent Technologies, cat #5065-9913) and separated by reversed-phase chromatography using a 12-cm column with an inner diameter of 75 μm, packed with 5 μm C18 particles (Nikkyo Technos Co., Ltd. Japan). Chromatographic gradients started at 97 % buffer A and 3 % buffer B with a flow rate of 300 nl/min, and gradually increased to 65% buffer A and 35% buffer B in 40 min. After each analysis, pre-column and column were washed for 10 min with 10% buffer A and 90% buffer B. Buffer A: 0.1% formic acid in water. Buffer B: 0.1% formic acid in acetonitrile.

The mass spectrometer was operated in positive ionization mode with nanospray voltage set at 2.5 kV and source temperature at 200 °C. Ultramark 1621 for the FT mass analyzer was used for external calibration prior the analyses. Moreover, an internal calibration was also performed using the background polysiloxane ion signal at m/z 445.1200. The instrument was operated in DDA mode and full MS scans with 1 micro scans at resolution of 60,000 were used over a mass range of m/z 350-2000 with detection in the Orbitrap. Auto gain control (AGC) was set to 1E6, and dynamic exclusion (60 seconds) and charge state filtering disqualifying singly charged peptides were both activated. In each cycle of DDA analysis, following each survey scan the top ten most intense ions with multiple charged ions above a threshold ion count of 5000 were selected for fragmentation at normalized collision energy of 35%. Fragment ion spectra produced via collision-induced dissociation (CID) were acquired in the ion trap, AGC was set to 5E4, isolation window of 2.0 m/z, activation time of 0.1 ms and maximum injection time of 100 ms was used. All data were acquired with Xcalibur software v2.2.

Proteome Discoverer software suite (v1.3.0.339, Thermo Fisher Scientific) and the Mascot search engine (v2.3, Matrix Science) [7] were used for peptide identification. The data were searched against an in-house generated database containing all proteins corresponding to *Caenorlabditis elegans* in the Swissprot database plus the most common contaminants previously described [8]. A precursor ion mass tolerance of 7 ppm at the MS1 level was used, and up to three miscleavages for trypsin were allowed. The fragment ion mass tolerance was set to 0.5 Da. Oxidation of methionine and protein acetylation at the N-terminal were defined as variable modification; whereas carbamidomethylation on cysteines was set as a fix modification. In all cases, false discovery rate (FDR) in peptide identification was evaluated by using a decoy database and it was set to a maximum of 5%.

**Sequences of primers used in this study.**

**Primers used in semiquantitative RT-PCR**

***rsr-2* FW** 5’-CGAGGTGAAATGCACCGAAT-3’

***rsr-2* REV** 5’-GCCATTTTTTCGGCTCAA -3’

***act-1* FW** 5’-TTGAGCACGGTATCGTCACCAACT-3’

***act-1* REV** 5’-TCAGCGGTGGTGGTGAAAGAGTAA-3’

***rpl-12* FW** 5’-GTTGCGTCGGAGGAGAAGTCG-3’

***rpl-12* REV** 5’-GATGATGTCGTGTGGGTGTTGTC-3’

***gld-1* FW** 5’-CGACAATGTTCCAGCGGATCGTT-3’

***gld-1* REV** 5’-CTTCGGGAACGTCAAATCACTTGC-3’

***fbf-1* FW** 5’-ATGGACCAATCAAAAATGCGC-3’

***fbf-1* REV** 5’-CTGGGCAATGATAAGGGTGG-3’

***tra-2* FW** 5’-GGCTGCTGGTGAAGAGCTTTTTG-3’

***tra-2* REV** 5’-CGAGAACTGCTGAATGGCCACC-3’

***prp-8* FW** 5’-TTGACAGAGCATCCAGATCC-3’

***prp-8* REV** 5’-ATGGAATTTGGACAATGACTCC-3’

***dpy-2* FW** 5’-ATGAAATCGCAAACGAGTGG-3’

***dpy-2* REV** 5’-CTCTTGAAATTGTGGTGAATCG-3’

***dpy-8* FW** 5’-TCACCCAGAATACGCTGACG-3’

***dpy-8* REV** 5’-TTCTTGCGCCATTTCCTCTCG-3’

**Primers used to generate GFP reporter constructs by Gateway cloning.**

**attB2r *rsr-2* genomic and 3’UTR**

5’-GGGGACAGCTTTCTTGTACAAAGTGGTTATGTACAATGGAATCGGACT-3’

**attB3 *rsr-2* genomic and 3’UTR**

5’-GGGGACAACTTTGTATAATAAAGTTGCCCAGTTTTCAGGAGATTCTTC-3’

**attB2r *rsr-2* 3’UTR**

5’-GGGGACAGCTTTCTTGTACAAAGTGGCCTTTTTTCTTGTGTTTTAT-3’

**attB3 *rsr-2* 3’UTR**

5’-GGGGACAACTTTGTATAATAAAGTTGCCCAGTTTTCAGGAGATTCTTC-3’

**Primers used in Real Time PCR.**

***rsr-2* FW** 5’-GAGCCGAAAAAATGGCTGG-3’

***rsr-2* REV** 5’-CCCAGAAAATGTGGTTTTTTAGGC-3’

***fog-1* FW** 5’-TGTGGGAACTGAACCGGTCCGAA-3’

***fog-1* REV** 5’-ACTGGCGACACGGAGCCTCT-3’

***gld-1* FW** 5’-GCTCATTCCGGCTCCCGAGG-3’

***gld-1* REV** 5’-ACACGAGCTGGGTTTGGCGA-3’

***gld-3* FW** 5’-AGCGCAAGGATTGCCTCTGCC-3’

***gld-3* REV** 5’-CGTGATCCCCGTTGTCACTGGTC-3’

***fem-3* FW** 5’-TGGCAAGGCGGAACGGGAAA-3’

***fem-3* REV** 5’-CGGATCCGGATTGGGTAAAAATTGTCG-3’

***fbf-1* FW** 5’-ACATGCCACACCCGGGCACT-3’

***fbf-1* REV** 5’-CGTCTTCCAGACACAGCATCACAGC-3’

***tra-2* FW** 5’-TGGGACGCAAATCGAAGTGGCT-3’

***tra-2* REV** 5’-GGCGGCGAGGAAACCAGCAA-3’

***glp-1* FW** 5’-ACCAGCCGACGAAATCCCTCTCC-3’

***glp-1* REV** 5’-TGGCAGCAAGCCAGTGCAGA-3’

***puf-8* FW** 5’-TGCGTGTCACCATCAGGAAGGATCT-3’

***puf-8* REV** 5’-GGATGAGTTCACGGCGCTGTTCT-3’

***fbf-2* FW** 5’-TGGCGCAGCATGAGACACCT-3’

***fbf-2* REV** 5’-GGGTTGGTGGCCGCGATGTAA-3’

***nos-3* FW** 5’-ACTCACGTGGACATGGTGGAGGA-3’

***nos-3* REV** 5’-TCGGAGGAAGTTTTTGTTGTCGTTGGA-3’

**Primers used to make *rsr-2 in situ* hybridization probes.**

***rsr-2* sense** 5’-ATCCCGGCGTTGTGGTGACT-3’

***rsr-2* antisense** 5’-GCAAGCGAGACGAAAAATCG-3’

**Primers used in ChIP-qPCR**

**C05G5.7 promoter FW** 5’-GAGTAATGTATCCATGGAGCCG-3’

**C05G5.7 promoter REV** 5’-AGCAGATGAGGTTCCCCTG-3’

**C05G5.7 CDS FW** 5’-CCAACATGCGTGTCGCCTA-3’

**C05G5.7 CDS REV** 5’-TAGGTCCAATCGAGGTAGATGC-3’

**F18E3.11 promoter FW** 5’-CTGTTTCCTCGAACCGAAGAACC-3’

**F18E3.11 promoter REV** 5’-ATCGACGATTCGAAGTGAGAATAGG-3’

**F18E3.11 CDS FW** 5’-ATGTCTCACGTTCTCGCCG-3’

**F18E3.11 CDS REV** 5’-CGGGGAGCATCTGATGATGT-3’

**F18E3.12 promoter FW** 5’-TTCAGACAATCGCCAGACAC-3’

**F18E3.12 promoter REV** 5’-TGGGACTCCGCCTATTTTCTG-3’

***phy-2* promoter FW** 5’-TGTCTACCTGCCTACAAGG-3’

***phy-2* promoter REV** 5’-ACACCGCCTGTCTATATGGG-3’

***phy-2* TSS FW** 5’-TCGCCACCGATTTTGACCTCG-3’

***phy-2* TSS REV** 5’-ATCTGCATGAGCAAGTCCTGCC-3’

***phy-2* intron 4 FW** 5’-TTCAAGGAAAGGATATGGTTGG-3’

***phy-2* intron 4 REV** 5’-ATCGTACTCAACTCTCTGC-3’

***phy-2* 3’UTR FW** 5’-ACTTTATTGGAGATTTGTCCCC-3’

***phy-2* 3’UTR REV** 5’-TTGGAACCGTGAAACACCAG-3’

***act-1* promoter FW** 5’-AGCTCACTCATCTCCACG-3’

***act-1* promoter REV** 5’-TCTGGTGTTATCTGTTCGC-3’

**References for expanded material and methods:**

1. Duerr JS (2006) Immunohistochemistry. WormBook: 1-61.

2. Zhong M, Niu W, Lu ZJ, Sarov M, Murray JI, et al. (2010) Genome-wide identification of binding sites defines distinct functions for Caenorhabditis elegans PHA-4/FOXA in development and environmental response. PLoS Genet 6: e1000848.

3. Blankenberg D, Von Kuster G, Coraor N, Ananda G, Lazarus R, et al. (2010) Galaxy: a web-based genome analysis tool for experimentalists. Curr Protoc Mol Biol Chapter 19: Unit 19 10 11-21.

4. Zhang Y, Liu T, Meyer CA, Eeckhoute J, Johnson DS, et al. (2008) Model-based analysis of ChIP-Seq (MACS). Genome Biol 9: R137.

5. Chevallet M, Luche S, Rabilloud T (2006) Silver staining of proteins in polyacrylamide gels. Nat Protoc 1: 1852-1858.

6. Rappsilber J, Mann M, Ishihama Y (2007) Protocol for micro-purification, enrichment, pre-fractionation and storage of peptides for proteomics using StageTips. Nat Protoc 2: 1896-1906.

7. Perkins DN, Pappin DJ, Creasy DM, Cottrell JS (1999) Probability-based protein identification by searching sequence databases using mass spectrometry data. Electrophoresis 20: 3551-3567.

8. Bunkenborg J, Garcia GE, Paz MI, Andersen JS, Molina H (2010) The minotaur proteome: avoiding cross-species identifications deriving from bovine serum in cell culture models. Proteomics 10: 3040-3044.
