# Supplementary material for: RSR-2, the Caenorhabditis elegans Ortholog of Human Spliceosomal Component SRm300/SRRM2, Regulates Development by Influencing the Transcriptional Machinery
Source: PLoS Genet. 2013 Jun 6;9(6):e1003543. doi: 10.1371/journal.pgen.1003543 (PMC3675011; doi:10.1371/journal.pgen.1003543)
Supplement: Text S2 — Supplementary information for Supporting Figure S3. (DOCX) [file pgen.1003543.s016.docx]

**TEXT S2.** Supplementary information for Supporting Figure 3.

There are two main cell fate decisions during the *C. elegans* germline development: (i) the transformation of proliferating cells into meiotic cells and (ii) the switch from spermatogenesis to oogenesis. If this mitosis/meiosis switch, which is regulated by the GLP-1/Notch signaling pathway, fails, cells do not enter into meiosis and keep proliferating forming a tumorous germline. In *rrf-1(pk1417); glp-1(oz264)* animals grown at 20°C, negative control *gfp(RNAi)*, positive control *lsm-2(RNAi)* and *rsr-2(RNAi)* presented tumorous germline in 14.5%, 31.5%, and 15.6% of the gonads respectively. These results indicate that *rsr-2(RNAi)* does not affect the meiotic entry decision.

Since overproliferation in the germline could be also a consequence of a defect in meiotic progression, we performed similar RNAi assays with the strain *rrf-1(pk1417); gld-3(q730)*. These experiments exclude a major role of *rsr-2* in meiotic progression. Importantly we observed the Mog phenotype (~ 40% at 20 ˚C) among the non-tumorous *rsr-2(RNAi)* animals, confirming the effect of the *rsr-2* RNAi.

Additionally, we detected the phosphorylation of Histone 3 by immunofluorescence and calculated the mitotic index of *gfp(RNAi)* and *rsr-2(RNAi)* germlines at two conditions. Neither one-day adults grown at 15 ˚C nor L4 grown at 25˚C showed increased mitotic index in *rsr-2(RNAi)* animals (data not shown).
